# Guanidino acid hydrolysis by the human enzyme annotated as agmatinase

**DOI:** 10.1038/s41598-022-26655-4

**Published:** 2022-12-21

**Authors:** Malte Sinn, Marco Stanoppi, Franziskus Hauth, Jennifer R. Fleming, Dietmar Funck, Olga Mayans, Jörg S. Hartig

**Affiliations:** 1 Department of Chemistry, University of Konstanz, Konstanz, Germany; 2 Konstanz Research School Chemical Biology (KoRS-CB), University of Konstanz, Konstanz, Germany; 3 Department of Biology, University of Konstanz, Konstanz, Germany

**Keywords:** Enzymes, Neurochemistry, Biomarkers, Hydrolases

## Abstract

Guanidino acids such as taurocyamine, guanidinobutyrate, guanidinopropionate, and guanidinoacetate have been detected in humans. However, except for guanidionacetate, which is a precursor of creatine, their metabolism and potential functions remain poorly understood. Agmatine has received considerable attention as a potential neurotransmitter and the human enzyme so far annotated as agmatinase (AGMAT) has been proposed as an important modulator of agmatine levels. However, conclusive evidence for the assigned enzymatic activity is lacking. Here we show that AGMAT hydrolyzed a range of linear guanidino acids but was virtually inactive with agmatine. Structural modelling and direct biochemical assays indicated that two naturally occurring variants differ in their substrate preferences. A negatively charged group in the substrate at the end opposing the guanidine moiety was essential for efficient catalysis, explaining why agmatine was not hydrolyzed. We suggest to rename AGMAT as guanidino acid hydrolase (GDAH). Additionally, we demonstrate that the GDAH substrates taurocyamine, guanidinobutyrate and guanidinopropionate were produced by human glycine amidinotransferase (GATM). The presented findings show for the first time an enzymatic activity for GDAH/AGMAT. Since agmatine has frequently been proposed as an endogenous neurotransmitter, the current findings clarify important aspects of the metabolism of agmatine and guanidino acid derivatives in humans.

## Introduction

The human protein currently annotated as agmatinase (AGMAT) belongs to the arginase or ureohydrolase superfamily. Most members of this family catalyze the hydrolysis of a guanidine group to urea and the respective amines in metabolites such as arginine^1^, agmatine^2^ and guanidinobutyrate (GBA)^3^. In the active site of these enzymes, two metal ions, typically Mn^2+^, are coordinated by highly conserved Asp and His residues. The di-metal center positions a hydroxide ion for the nucleophilic attack at the guanidine moiety^4^. AGMAT was proposed to be an agmatinase catalyzing the hydrolysis of agmatine to putrescine and urea. However, the designation of the enzymatic activity relied on indirect evidence by complementation of an agmatinase-deficient yeast strain with the human candidate gene^5^ and a biochemical study that lacked demonstration of activity using a purified enzyme^6^. It has been noted before that AGMAT activity has never been demonstrated conclusively and it was speculated by others that so-far unidentified co-factors might be necessary for its activity^7^. For the recently discovered guanidine hydrolase, also a member of the ureohydrolase family, we showed that its activity depends on the chaperone-mediated insertion of Ni^2+^ rather than Mn^2+^ into the active site^8^. It is conceivable that isolated or recombinant AGMAT similarly lacks essential factors for its activity. However, as we show with the present work, purified recombinant AGMAT was active when the appropriate substrates were provided.

Arginine is by far the most abundant guanidine compound and to current knowledge the direct or indirect precursor of all other linear guanidine derivatives. Apart from its role as proteinogenic amino acid, arginine serves as the precursor for the generation of urea, nitric oxide, polyamines, and creatine. In creatine biosynthesis, the intermediate guanidinoacetate is formed from glycine and arginine by mitochondrial glycine amidinotransferase (GATM)^9^. Creatine, a bi-substituted guanidine, is used as transitory acceptor of high-energy phosphates in vertebrates with creatine kinases mediating the reversible transfer of a phosphate group between ATP and creatine^10^. In some lower animals, homologs of creatine kinases use alternative guanidine derivatives such as taurocyamine as phosphate acceptor^11^. The fundamental roles of the mentioned metabolites in human physiology depend on the presence of the guanidine moiety and many of the producing and degrading enzymes are well characterized.

Besides arginine, guanidinoacetate, and creatine, a range of other linear guanidine derivatives have been detected in the human brain^12^ and in body fluids such as urine, saliva and blood plasma^13–15^. However, the metabolism and potential physiological roles of guanidine derivatives such as agmatine, taurocyamine (TC), GBA and guanidinopropionate (GPA) are only poorly understood in humans. Agmatine was detected in the human brain^16^, and many studies have since linked agmatine to neuroprotection or nerve regeneration, and proposed its use as a potential therapeutic for neuropathies^17^. Agmatine blocks nicotinic receptors^18^ and binds to imidazoline and α_2_-adrenergic receptors^16^ and has been suggested to function as an endogenous neurotransmitter^19^.

Only two years after the discovery of agmatine in the vertebrate brain, an agmatinase activity was reported in the rat brain^20^. This activity is mediated by LIM and calponin homology domains-containing protein 1 (LIMCH1), also named agmatinase-like protein (ALP) which also depends on Mn^2+^ but is not a member of the ureohydrolase family^21^. These findings raised the question whether agmatine is metabolized in humans by LIMCH1 or by AGMAT. The AGMAT homologs in mouse, rat and related rodents lack some of the conserved residues for Mn^2+^ coordination and are therefore considered to be catalytically inactive^22^.

Far less is known about the functions of the additional guanidine derivatives TC, GBA and GPA that have been detected in humans. Generally, concentrations of all kinds of guanidine compounds were higher in patients with renal failure compared to healthy subjects^14^. GPA acted antihyperglycemic^23^ by the induction of GLUT4 expression through reduced ATP, creatine and phosphocreatine levels^24^. Guanidine derivatives were reported to have various effects on the central nervous system. For example, increased concentrations of a range of guanidines have been related to convulsions^25,26^. GPA has been reported to depolarize neurons^27^ and TC interacts with neuroreceptors^28–31^. These findings demonstrate the general interest in mechanisms that modulate the levels of guanidine derivatives in human physiology.

Considering that AGMAT had been annotated as an agmatinase despite an apparent lack of a demonstration of agmatine hydrolysis by AGMAT, we set out to investigate the reactions catalyzed by this enzyme. When we compared AGMAT to other members of the ureohydrolase family, we observed the highest sequence similarity to guanidinobutyrase (GbuA) from *Pseudomonas aeruginosa*^3^. We demonstrate here that the enzyme efficiently hydrolyzed linear guanidino acids such as TC and GBA, whereas GPA and arginine were turned over less efficiently and guanidinoacetate or compounds lacking acidic residues such as agmatine were not accepted as substrates. Modeling of the 3D-structure of AGMAT revealed that two naturally occurring variants of the human enzyme differ in a residue (R/G105) forming part of the substrate-binding pocket. We characterized both variants with regard to their activity towards a broad panel of guanidine-containing compounds and found slightly different substrate specificities. Apart from being present in human diets^32,33^ and potentially produced by the human microbiome^34^, we further show that the identified guanidino acid substrates were produced by the promiscuous action of human glycine amidinotransferase. Taken together, the discovery of a guanidino acid hydrolase activity revises some fundamental aspects of the metabolism of guanidine-containing compounds in humans.

## Results

### Human AGMAT efficiently hydrolyzes guanidino acids

**Figure 1:**
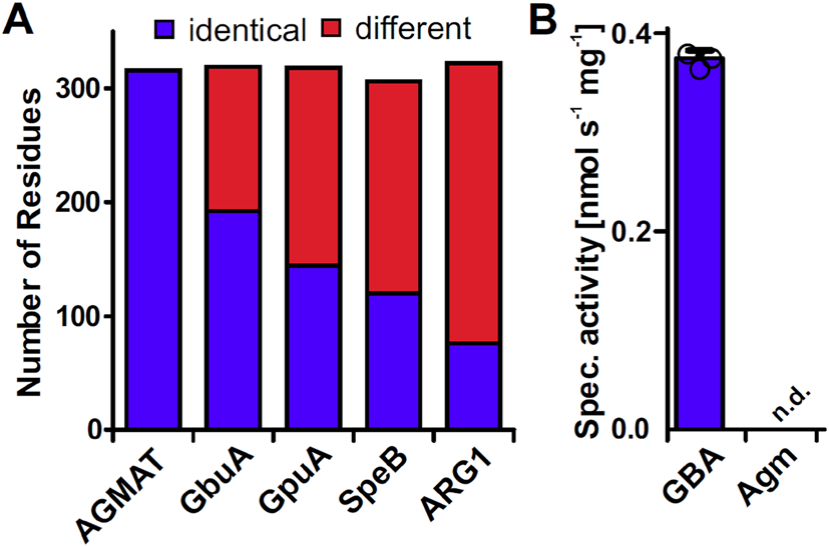
Sequence comparison and catalytic activity of human AGMAT variant R105. (**A**) Amino acid identity of the mature form of AGMAT compared to guandidinobutyrase (GbuA) and guanidinopropionase (GpuA) from *Pseudomonas aeruginosa*, agmatinase from *Escherichia coli* (SpeB) and human arginase 1 (ARG1). The numbers of identical (blue) and different (red) residues of the respective enzymes in comparison to AGMAT were derived from a multiple sequence alignment (Supplementary Fig. S1). (**B**) Specific activity of recombinantly expressed and purified AGMAT in the presence of 10 mM GBA or agmatine (Agm). Column represents the average of technical triplicates and consistent results were obtained with independent preparations (Data from Fig. 2C). (n.d. = not detected; limit of detection ~ 0.02 nmol s^-1^ mg^-1^), error bars, s.d.

Sequence comparison revealed that the mature form of AGMAT, obtained after removal of the predicted mitochondrial transit peptide (35 amino acids), shared 39.1% identical amino acids with the functionally characterized agmatinase (SpeB) from *E. coli* and 35.8% with human arginase (ARG1, Fig. 1A and Supplementary Fig. S1)*.* However, two loops that were reported to mediate substrate specificity of SpeB and Arg1^35^ show no conservation between AGMAT and SpeB or Arg1. Furthermore, we noted that guanidinobutyrase GbuA and guanidinopropionase GpuA from *Pseudomonas aeruginosa*^3,36^ share considerably higher degrees of similarity with 60.2% and 45.2% identical amino acids, respectively with AGMAT. Since the activity of human AGMAT has never been demonstrated conclusively, we tested whether guanidinobutyrate is hydrolyzed more efficiently compared to the currently assumed substrate agmatine. In accordance with the activity of the more similar enzyme, we observed that recombinant AGMAT catalyzed the hydrolysis of GBA whereas agmatine hydrolysis was not detected (Fig. 1B).

**Figure 2:**
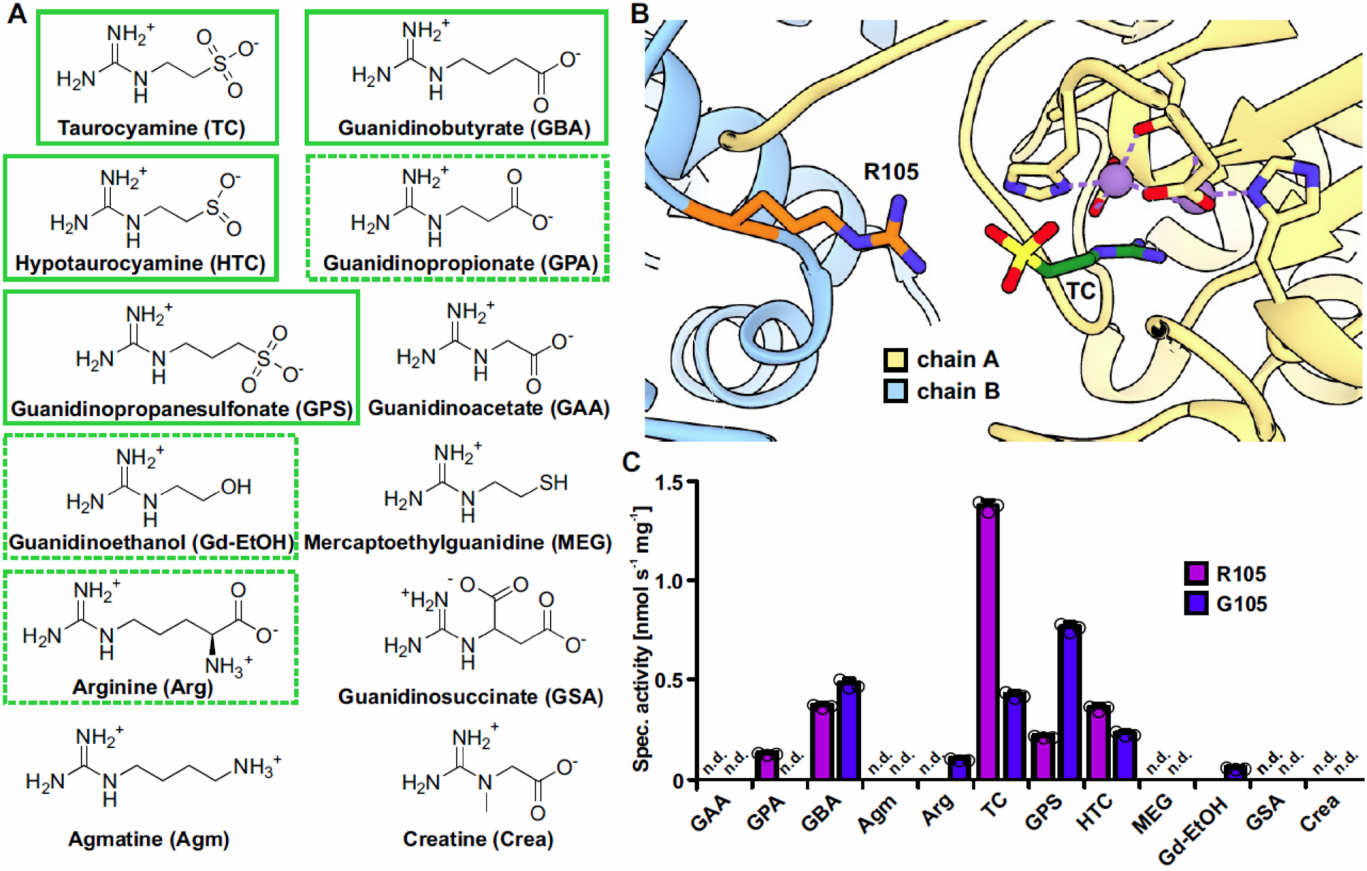
Structural model and substrate specificity of GDAH/AGMAT. (**A**) Structures of guanidine derivatives tested as substrates for GDAH. Boxed substrates were hydrolyzed by both variants of GDAH, whereas substrates in a dashed box were only hydrolyzed by either the R105 or the G105 variant. (**B**) Close-up of the substrate binding pocket of a GDAH model showing the position of R105 from the neighboring subunit in relation to the substrate taurocyamine (TC). A global view of the two subunits is provided in Supplementary Fig. S2. Details about the modelling and substrate docking are provided in the main text. (**C**) Specific activities of the two GDAH variants recombinantly expressed in *E. coli* with various substrates. Columns represent the average of technical triplicates and consistent results were obtained with independent preparations. (n.d. = not detected; limit of detection ~ 0.02 nmol s^−1^ mg^−1^), error bars, s.d. For the sensitivity of the colorimetric assay, see the urea calibration curve (Supplementary Fig. S3).

The surprising discovery of guanidinobutyrate as substrate for the human agmatinase prompted us to test a variety of different substrates. We found that guanidino acids of a certain chain length such as guanidinobutyrate and taurocyamine are generally accepted as substrates for AGMAT (Fig. 2A). In order to gain structural insights into the substrate preference of AGMAT, we built a model of the 3D-structure of a monomer using the Robetta server^37^. Members of the arginase or ureohydrolase family commonly form hexamers, where a neighboring subunit contributes to the formation of the substrate-binding groove in another subunit leading to a shared active site. To properly assess the selectivity of the enzyme and the features of the AGMAT active site, we modeled the local quaternary structure of the enzyme based on the arrangement of subunits in the crystal structure of GbuA (PDB entry 3NIO)^36^ (Fig. 2B). For this, the catalytic residues of AGMAT and GbuA were superimposed to match two neighboring subunits as present in the biological assembly of GbuA. Ions, which are also not included in Robetta models, were placed according to their location relative to the active site residues in GbuA. The residues coordinating two Mn^2+^ in the active site as well as His201 and Glu320 essential for catalytic activity in human arginase^38^ are fully conserved between GbuA and AGMAT. In addition, eight out of 10 residues in close proximity to the substrate-binding pocket are conserved between AGMAT and GbuA (Supplementary Fig. S1). Due to this high similarity, GbuA is an excellent template to evaluate the active site features of AGMAT. Within the GbuA structure, it can be seen that the active site of a subunit is capped by residue R72 from a neighboring subunit, so that this residue could interact with the carboxylate group of the substrate guanidinobutyrate. Interestingly, in the equivalent position to R72 in AGMAT (position 105) two variants exist, with either arginine or glycine in this position. In the genomes covered by the NCBI short genetic variation database, the G105 variant is slightly more abundant (57.7%) in the sampled human population compared to the R105 variant (42.3%) (dbSNP, entry rs6429757). Both variants have been used independently in studies aiming to demonstrate agmatinase activity of AGMAT^5,6^. To assess the possible influence of having a large, positively charged R105 residue instead of a small glycine, R105 was modeled using the rotamer observed in GbuA and taurocyamine, the best substrate in initial experiments, was docked into place in the active site using Autodock Vina. This complete substrate-bound AGMAT model shows that R105 can indeed reach into the active site of the neighboring subunit and potentially interacts with the sulfonic group of taurocyamine (Fig. 2B).

To compare the enzymatic activity and substrate specificity of both AGMAT variants (R105 and G105), we expressed the mature forms of the mitochondrial enzyme lacking the predicted N-terminal transit peptide of 35 amino acids (MitoFates^39^) in *E. coli* and purified them by Ni-affinity chromatography (Supplementary Fig. S1). Consistent with previous studies, we observed no agmatine hydrolysis with both variants of purified AGMAT. In contrast, both variants efficiently hydrolized GBA (Fig. 2A,C). When we provided a range of similar guanidine derivatives that differ in chain length and additional substitutions at a substrate concentration of 10 mM, both AGMAT variants additionally hydrolyzed TC, hypotaurocyamine (HTC, a reduced form of TC), and guanidinopropanesulfonate (GPS). For variant R105, the specific activity was three times higher for TC (1.4 nmol s^−1^ mg^−1^) compared to the second-best substrate GBA (0.4 nmol s^−1^ mg^−1^), whereas in comparison variant G105 showed reduced activity towards TC and slightly enhanced activity for GBA (Fig. 2C). As described above, when TC is placed into the structural model, its sulfonate group is in proximity of the guanidine group of R105 (Fig. 2B). Variant G105 hydrolyzed arginine to a small extent, whereas variant R105 was inactive with arginine but hydrolyzed GPA instead (Fig. 2C). Urea release from agmatine or other guanidine derivatives lacking a negatively charged group (mercaptoethylguanidine, methylguanidine, guanidine) was not detected, except for guanidinoethanol that was a poor substrate for variant G105. Similarly, guanidinosuccinate, guanidinoacetate and creatine were not hydrolyzed by either variant of AGMAT at a substrate concentration of 10 mM. Taken together, AGMAT exhibits a rather broad substrate specificity towards linear guanidino acids with intermediate chain lengths, but is almost strictly dependent on a negatively charged head group. Variant R105 prefers shorter substrates compared to variant G105 that even showed weak activity with arginine. In light of our results, we propose to re-annotate the human enzyme formerly known as agmatinase (AGMAT) as guanidino acid hydrolase (GDAH) to better reflect its enzymatic activity.

**Figure 3:**
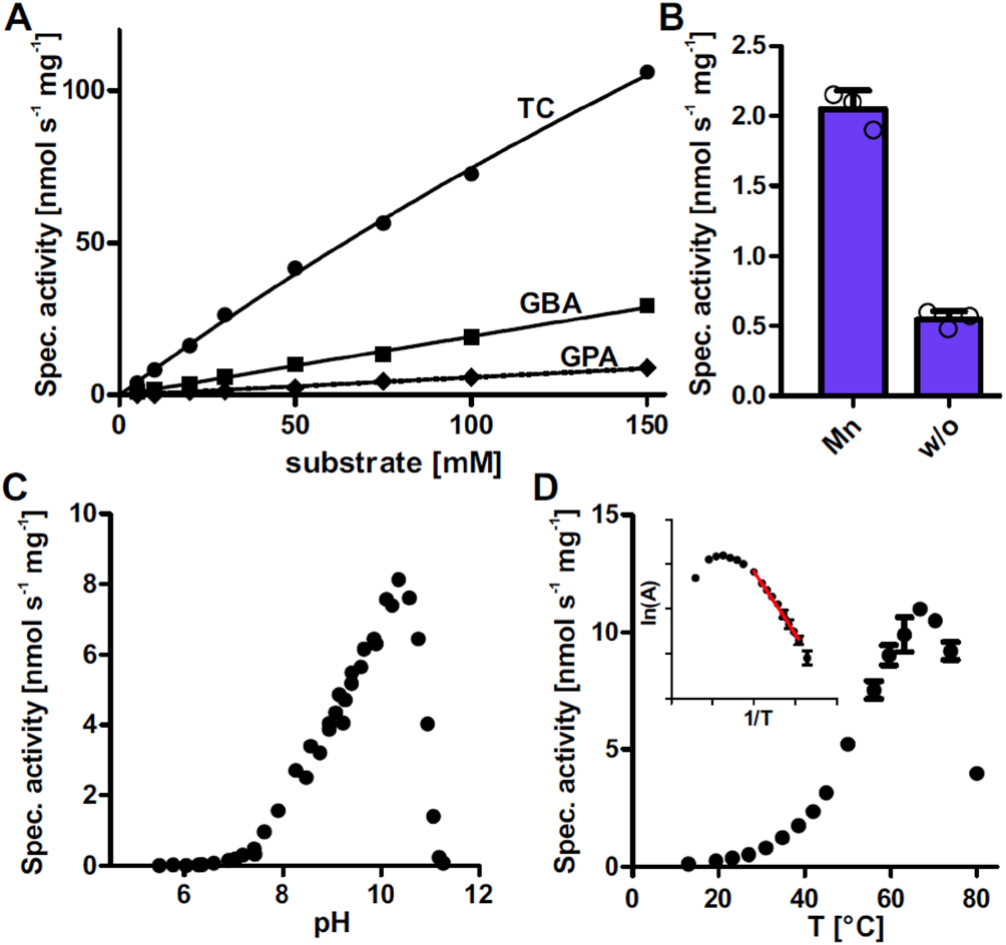
Kinetic characterization of GDAH variant R105. (**A**) The specific activity of GDAH variant R105 was determined for different substrate concentrations of TC, GBA and GPA. Means of triplicates were plotted against the concentration with error bars representing the SD. Curves were fitted with Michaelis–Menten kinetics. (**B**) Activity of purified GDAH expressed in medium supplemented with 0.5 mM Mn^2+^ (Mn) or without supplemental Mn^2+^ (w/o). Columns represent the average of technical triplicates and consistent results were obtained with independent preparations. Error bars, s.d. (**C**) For the determination of the pH optimum of GDAH, specific activity of GDAH is plotted against the pH. GDAH was incubated with 10 mM TC in pH ranges from 5.5 to 11.5 and the specific activity was determined. (**D**) The temperature dependency of GDAH was assessed by determining the specific activity at 13 °C to 80 °C with 10 mM TC as substrate. Means of triplicates are plotted against the temperature. Inlet shows the Arrhenius-plot for the determination of the activation energy. The linear range of the Arrhenius-plot was used for fitting (red line).

Of the identified substrates of GDAH, only TC, GBA and GPA have been detected in human samples according to the Human Metabolome Database (HMDB)^40^. For this reason, we determined the concentration dependence of GDAH activity for these three compounds. The Michaelis constants (*K*_*M*_) for TC, GBA and GPA hydrolysis by GDAH were all > 50 mM for both, the GDAH variant R105 (Fig. 3A) and the variant G105 (Supplementary Fig. S1). The exact values could not be determined because no saturation was achieved within the concentration limits set by the solubility of the compounds. Throughout the concentration range tested, the specific activity for TC hydrolysis was approximately three times higher compared to GBA hydrolysis with variant R105, and GPA hydrolysis rates were substantially lower. GDAH was further characterized by using variant R105 with TC as best substrate. When GDAH was expressed in normal LB medium, its specific activity was low but the specific activity was increased four-fold when the protein was expressed in medium supplemented with 0.5 mM Mn^2+^ (Fig. 3B), consistent with a dependence on Mn^2+^ common to most members of the ureohydrolase family. The apparent pH optimum of GDAH was 10 and the apparent temperature optimum 67 °C (Fig. 3C,D). We determined the activation energy for TC hydrolysis to be 46 ± 1 kJ/mol (Fig. 3D).

### GDAH substrates are not used as alternative phosphagens

**Figure 4:**
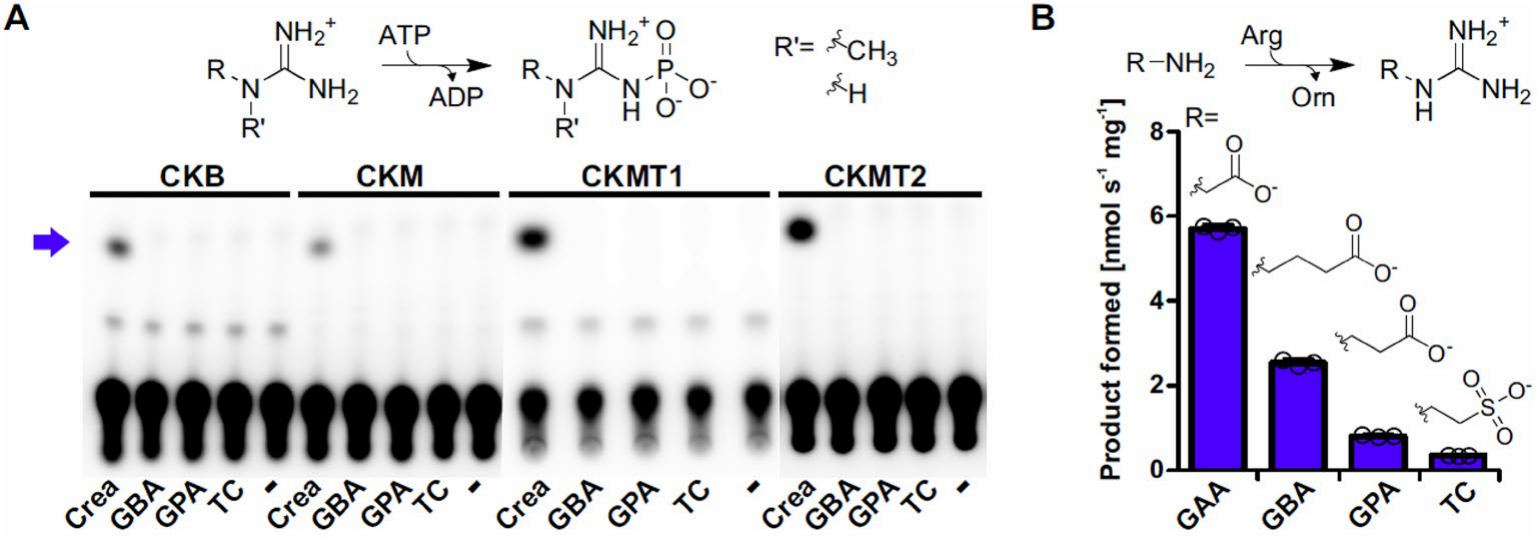
Formation of guanidino acids by GATM and their potential as phosphagens. **(A**) Radiograph of the phosphorylation reaction of four different human creatine kinase isoforms. Recombinant human creatine kinases CKB, CKM, CKMT1 and CKMT2 were incubated with γ-^32^P-ATP and 10 mM of the respective substrate (creatine (crea), GBA, GPA, TC and no substrate (-)). Reaction products were separated by TLC with Silica 60 as stationary phase. A phosphorylation product was only observed for creatine (blue arrow) for all creatine kinase isoforms. (**B**) Reactions of human GATM with its main substrate glycine in comparison to GABA, β-alanine and taurine forming the canonical GATM product guanidinoacetic acid (GAA) or the guanidino acids GBA, GPA and TC, respectively. Columns represent the average of technical triplicates and consistent results were obtained with independent preparations. Error bars, s.d.

Various guanidine derivatives ranging from arginine to creatine are used as phosphagens in different organisms, serving as cellular buffers of high energy phosphates. Phosphorylation of the guanidine group of TC yields phosphotaurocyamine, which is used as phosphagen in marine annelids and some protists^41,42^. In order to shed light on potential physiological roles of the identified GDAH substrates TC, GBA and GPA we tested whether these guanidine derivatives could serve as so far unrecognized phosphagens in humans by getting phosphorylated by one of the human creatine kinase isoforms. For this purpose, we overexpressed and purified the four charaterized human creatine kinase isoenzymes^43,44^. The creatine kinases were incubated with γ-^32^P-ATP as phosphate donor and creatine, TC, GBA, or GPA as substrates. Analysis of the reaction products by thin-layer chromatography revealed that all four creatine kinases accepted exclusively creatine as substrate (Fig. 4A). As a control we overexpressed and purified the TC kinase from *Arenicola brasiliensis*^41^. With this enzyme we observed a phosphorylation product specifically with TC as substrate under the same assay conditions utilized for the creatine kinases (Supplementary Fig. S1). These results indicate that the physiological function of GDAH is not concerned with the modulation of the levels of potential alternative phosphagens.

### GATM synthesizes GDAH substrates

GBA, GPA and TC have been detected in human fluids but an endogenous source has so far not been identified. However, all three compounds have been shown previously to be generated in side reactions of mitochondrial glycine amidinotransferase in rats from the respective amino homologs γ-aminobutyric acid (GABA), β-alanine and taurine^45^. In order to clarify whether the identified substrates for GDAH could be produced by an endogenous activity, we overexpressed and purified human mitochondrial glycine amidino transferase (GATM) lacking the mitochondrial transit peptide (uniprot entry: P50440) and performed activity assays with the potential substrates. Similar to the previously characterized enzyme from rat, human GATM had the highest activity with glycine, but also GABA, β-alanine and taurine were accepted as substrates with decreasing preference (Fig. 4B). Both GATM and GDAH are mitochondrial enzymes especially expressed in kidney and liver (https://www.ncbi.nlm.nih.gov/gene/2628; https://www.ncbi.nlm.nih.gov/gene/79814).

## Discussion

20 years ago, a human gene from the ureohydrolase family has been annotated as agmatinase. However, we demonstrate that GDAH/AGMAT hydrolyzes guanidino acids rather than agmatine (Fig. 2C). Human GDAH has a high degree of sequence similarity with the guanidinobutyrase GbuA of *Pseudomonas aeroguinosa* and its three-dimensional structure can be predicted with high confidence based on the GbuA structure (PDB 3NIO). Two enzyme variants exist in humans in regard to residue 105 that likely participates in the binding of the substrates. In 57.7% of human sequences arginine is replaced by glycine at this position. Our modeling based on the *P. aeroguinosa* guanidinobutyrase and subsequent docking of TC suggests that R105 can extend into a neighboring subunit’s active site, positioning R105 to directly contact the negatively charged head group of the substrate. Although we observed residual arginine hydrolysis by GDAH variant G105, agmatine was not hydrolyzed by both tested GDAH variants. Instead, both GDAH variants hydrolyzed TC and GBA efficiently. Variant R105 exhibited the highest activity with TC, whereas variant G105 turned over GBA and TC to the same extent. Both GDAH variants also hydrolyzed GPA but for the G105 variant, this activity was only detectable at very high substrate concentrations far above the concentrations detected in human samples that are in the low µM range (HMDB)^40^. Still, we cannot exclude that concentrations might be higher in cells or certain compartments such as the mitochondria, where both GDAH and GATM are predicted to be localized (NCBI Gene database). Overall, variant G105 preferred longer substrates compared to variant R105 and even accepted arginine and guanidinoethanol as poor substrates. Both *K*_*M*_ and reaction rate for all substrates are representative of only low to intermediate enzymatic activity. However, kinetic constants in this range do not allow to draw conclusions about a potential physiological relevance. For example, we recently reported that despite its relatively high *K*_*M*_ the guanidine hydrolase GdmH allowed *Synechocystis* to utilize guanidine as sole nitrogen source^8^. The analysis of further substrates that are not known to occur in humans indicated that the activity of GDAH strictly depends on a negatively charged functionality opposite to the guanidine group. The first report of an enzymatic activity of this enzyme allowed a more thorough characterization: GDAH shares common features such as rather high *K*_M_ values, Mn^2+^-dependency, and high apparent temperature and pH optima with other members of the ureohydrolase superfamily^46^.

We show that GDAH substrates GBA, GPA and TC were not accepted as substrates of human creatine kinases but could be formed by the action of human GATM. GATM promiscuously transferred the amidino group of arginine to glycine, GABA, β-alanine and taurine yielding the respective guanidine compounds GBA, GPA and TC as it has been demonstrated for some mammalian homologs before^45,47^. Expression data and subcellular localization suggest that GDAH and GATM co-localize in the mitochondria of kidney, liver and brain. Even if taurine is a minor substrate of GATM, TC could be formed in substantial amounts via this reaction as taurine is produced in the liver and reaches concentrations of 10–50 mM^48^. In addition to the activity of GATM, the identified guanidino acids could be produced by so-far unknown metabolic processes. GBA could be an intermediate of agmatine/arginine catabolism via guanidinobutyraldehyde^49^. However, the activity of diamine oxidase towards agmatine to produce guanidinobutyraldehyde is controversial^50^. Alternatively, GBA, GPA and TC could originate from direct uptake from the diet^32,33^ or from gut microbial activities^34^.

TC has been used since the late 1970’s to manipulate taurine levels in different organs or tissues since it is a competitive inhibitor of the taurine transporter TAUT^51,52^. Additionally, TC has been reported as an antagonist of GABA_A_ and glycine receptors^28–31^. GBA has been detected in the brain, liver and kidney of vertebrates and insects and elevated levels of GBA and TC in the brain of rabbits have been related to convulsions^25,26^. Given these effects of the identified GDAH substrates, a possible function of GDAH could be the hydrolysis of potentially deleterious concentrations of guanidino acids as a means of detoxification. This hypothesis would also agree with the observed broad substrate specificity of GDAH. GDAH expression has been reported to be reduced in diabetic patients with breast cancer or clear cell type of renal cell carcinoma^53,54^. GDAH variant R105 of GDAH has been linked to type 1 diabetes^55^. On the other hand, GDAH was reported to promote lung adenocarcinoma tumorigenesis and the progression of colorectal cancer by inducing chronic inflammation^56,57^.

The potential involvement of GDAH/AGMAT in agmatine metabolism has received increasing attention from neuroscientists starting in 1994 when agmatine had been detected in the human brain^16^. Agmatine is discussed to have beneficial effects on disorders of the central nervous system like depression, nerve regeneration and epilepsy among others and is generally believed to be neuroprotective^7,17^. GDAH is highly expressed in kidney, liver and hippocampal interneurons^58–60^. The presented findings implicate that GDAH is unlikely to contribute directly to the modulation of endogenous agmatine concentrations. Further evidence for a physiological role of GDAH as guanidino acid hydrolase comes from two recent observations: Concentrations of GBA and GPA have been found to negatively correlate with the expression level of GDAH in a genome-wide study of genes affecting metabolite concentrations in saliva^15^. In addition, a further study identified an association of altered concentrations of GPA with genetic variations in the chromosomal locus of *GDAH*^61^. In conclusion, the findings presented in this study revise some fundamental aspects of the metabolism of guanidine derivatives in humans and pose further questions regarding the functional or pathological roles of GDAH and its substrates in human physiology.

## Methods

### Bacterial cultivation, cloning, protein overexpression and purification

*E. coli* BL21(λDE3) gold (Invitrogen) or *E. coli* SoluBL21 (Genlantis) were grown in LB with 50 µg/ml kanamycin when transformed. Genes of interest (human GDAH, GATM, CKM, CKB, CKMT1, and CKMT2, as well as TCK from *Arenicola brasiliensis*^41^) were codon optimized for *E. coli* and synthesized by GeneArt (LifeTechnologies). Mitochondrial target sequences were predicted with MitoFates^39^ and excluded to obtain mature enzymes. DNA constructs were directly cloned into a pET24 derivative by Gibson assembly to obtain enzymes with an N-terminal 6xHis-tag followed by a TEV cleavage site. For recombinant protein expression, *E. coli* BL21(λDE3) gold (Invitrogen) or *E. coli* SoluBL21 (Genlantis), in case of GDAH and CKMT1, were transformed with the expression constructs, grown in LB supplemented with 0.5 mM MnCl_2_, if not stated otherwise, at 37 °C to an OD_600_ of 0.6, transferred to 18 °C and induced over night with 1 mM IPTG. The cells were harvested by centrifugation, resuspended in enzyme buffer (50 mM Tris–HCl pH 8, 100 mM NaCl) supplemented with 1 × EDTA-free cOmplete protease inhibitor (Roche) and lysed by ultrasonication (Branson). After centrifugation at 12,000 g for 20 min at 4 °C, the soluble protein fraction was incubated with His-bind NiNTA resin (Qiagen, Hilden, Germany) for 30 min at 4 °C. The resin was loaded into gravity flow columns and washed sequentially with enzyme buffer supplemented with 20 mM or 50 mM imidazole. The His-tagged enzymes were eluted in enzyme buffer supplemented with 500 mM imidazole and desalted into enzyme buffer by passage through PD50 columns (GE lifesciences).

### Modeling

A comparative model of AGMAT was produced using the Robetta server^37^. This produced a model containing just one subunit. Therefore, to reconstitute the oligomeric state, the AGMAT model was aligned to chain A of 3NIO using the catalytic site residues (H129, D152, H154, D156 and D243 of 3NIO). A second copy of the AGMAT model was then structurally aligned to chain B of 3NIO. The active site manganese ions were modeled by using the positions of the ions in 3NIO relative to the active site residues. In the produced model R105 was modeled to have the rotameric conformation of R72 in *Pa*GbuA. The AGMAT models were prepared for docking by removing the hydrogens from original Robetta models and re-adding and reparametrizing them using Chimera UCSF Dock Prep. Hydrogens and charges were calculated using ANTECHAMBER^62^. The AMBER ff14SB forcefield^63^ was applied for standard residues and Gasteiger for other residues^64^. Taurocyamine (TC) parameters (ZINC000004095756) were downloaded from ZINC15; https://zinc.docking.org/ and docked into the active site of AGMAT models using Autodock Vina 1.1.2^65^. A search box large enough to include the entirety of residue 105 was used. Nine binding modes were tested, with an exhaustiveness of eight and a maximum energy difference of 3 kcal/mol. A representative TC docking result where TC occupied the active site was chosen to assess the potential distance between the TC sulfate group and R105 from the neighboring subunit.

### Ureohydrolase assay

For the ureohydrolase activity assays, 4–8 µg of the purified enzyme was incubated at 30 °C with 10 mM substrate in a buffer containing 133 mM Tris–HCl, pH 8, 1 mM MnCl_2_ and 1 mM Na-maleate for 30 to 60 min. The reaction was terminated by the addition of 87% (v/v) acetic acid. For the kinetic analyses, substrate concentrations, buffer and pH, or temperature, were modified as indicated in the respective figures. Urea released by ureohydrolase activity was determined colorimetrically as described in reference^66^. In brief, 50 µl of enzyme assay were mixed with 100 µl of color reagent (62 mM butanedionmonoxime, 3.6 mM thiosemicarbazide) and 150 µl acid reagent (120 µM FeCl_3_ and 10 mM phosphoric acid in 20% (w/v) sulphuric acid) and incubated for 10 min at 96 °C. Samples with known concentrations of urea were treated in the same way and were used to establish a calibration curve. The absorption at 520 nm was measured in a 96-well plate (TECAN Spark Reader). Non-linear fitting of the data to Michaelis Menten kinetics {ν = (*V*_*max*_ [S])/(*K*_*M*_ + [S])} was performed with GraphPad Prism6.

### Glycine amidinotransferase assay

3.4 µg glycine amidinotransferase was incubated in a buffer containing 80 mM KCl, 20 mM NaCl, 2 mM MgCl_2_, 80 mM Tris (pH 8) with 10 mM arginine and 10 mM of the indicated substrate. The ornithine concentration was measured as described^67,68^. In brief, 100 µl sample were mixed with equal volumes of both ninhydrin solution (25 mg ml^-1^ ninhydrin in 60% (v/v) acetic acid and 13.8% (w/v) phosphoric acid) and glacial acetic acid. The reaction mixture was incubated for 15 min at 96 °C. Reactions were chilled and the absorption at 520 nm was measured in a 96-well plate (TECAN Spark Reader).

### Creatine kinase assay

5 µg of the respective creatine or TC kinase isoform was incubated with 10 mM creatine, GBA,GPA or TC and 1 mM ATP and 1 µCi γ-^32^P-ATP in 100 µl buffer containing 80 mM KCl, 20 mM NaCl, 2 mM MgCl_2_, 80 mM Tris (pH 8) for 1 h at 30 °C. The reaction mixture was separated by TLC with silica gel 60 (Merck) as stationary phase and isopropanol: 25% NH_3_: H_2_O (3:1:1) as mobile phase. Radiographs were recorded with a Typhoon FLA 7000 laser scanner (GE Healthcare).

### Non-commercial guanidine compounds

Synthesis and characterization of commercially unavailable guanidino compounds is described in the supplementary information.

## Supplementary Information

Supplementary Information.

## Data Availability

The raw data are presented in the manuscript and are available from the corresponding authors upon reasonable request.

## References

[1] Kanyo ZF, Scolnick LR, Ash DE, Christianson DW (1996). Structure of a unique binuclear manganese cluster in arginase. Nature.

[2] Perozich J, Hempel J, Morris SM (1998). Roles of conserved residues in the arginase family. Biochim. Biophys. Acta.

[3] Nakada Y, Itoh Y (2002). Characterization and regulation of the gbuA gene, encoding guanidinobutyrase in the arginine dehydrogenase pathway of *Pseudomonas aeruginosa* PAO1. J. Bacteriol..

[4] Uribe E (2020). Functional analysis of the Mn(2+) requirement in the catalysis of ureohydrolases arginase and agmatinase—A historical perspective. J. Inorg. Biochem..

[5] Mistry SK (2002). Cloning of human agmatinase. An alternate path for polyamine synthesis induced in liver by hepatitis B virus. Am. J. Physiol. Gastrointest. Liver Physiol..

[6] Iyer RK, Kim HK, Tsoa RW, Grody WW, Cederbaum SD (2002). Cloning and characterization of human agmatinase. Mol. Genet. Metab..

[7] Laube G, Bernstein HG (2017). Agmatine: Multifunctional arginine metabolite and magic bullet in clinical neuroscience?. Biochem. J..

[8] Funck D (2022). Discovery of a Ni(2+)-dependent guanidine hydrolase in bacteria. Nature.

[9] Wyss M, Kaddurah-Daouk R (2000). Creatine and creatinine metabolism. Physiol. Rev..

[10] Bessman SP, Carpenter CL (1985). The creatine-creatine phosphate energy shuttle. Annu. Rev. Biochem..

[11] Ellington WR (2001). Evolution and physiological roles of phosphagen systems. Annu. Rev. Physiol..

[12] Marescau B, Deyn P, Wiechert P, Gorp L, Lowenthal A (1986). Comparative Study of guanidino compounds in serum and brain of mouse, rat, rabbit, and man. J. Neurochem..

[13] Kai M, Miyazaki T, Ohkura Y (1984). High-performance liquid chromatographic measurement of guanidino compounds of clinical importance in human urine and serum by pre-column fluorescence derivatization using benzoin. J. Chromatogr..

[14] Tanaka A, Takahashi Y, Mizokuchi M, Shimada N, Koide H (1999). Plasma, urinary and erythrocyte concentrations of guanidino compounds in patients with chronic renal failure. Ren. Fail..

[15] Nag A (2020). Genome-wide scan identifies novel genetic loci regulating salivary metabolite levels. Hum. Mol. Genet..

[16] Li G (1994). Agmatine: An endogenous clonidine-displacing substance in the brain. Science.

[17] Piletz JE (2013). Agmatine: Clinical applications after 100 years in translation. Drug Discov. Today.

[18] Loring RH (1990). Agmatine acts as an antagonist of neuronal nicotinic receptors. Br. J. Pharmacol..

[19] Reis DJ, Regunathan S (2000). Is agmatine a novel neurotransmitter in brain?. Trends Pharmacol. Sci..

[20] Sastre M, Regunathan S, Galea E, Reis DJ (1996). Agmatinase activity in rat brain: A metabolic pathway for the degradation of agmatine. J. Neurochem..

[21] Uribe E, Salas M, Enriquez S, Orellana MS, Carvajal N (2007). Cloning and functional expression of a rodent brain cDNA encoding a novel protein with agmatinase activity, but not belonging to the arginase family. Arch. Biochem. Biophys..

[22] Morris SM (2003). Vertebrate agmatinases: What role do they play in agmatine catabolism?. Ann. N. Y. Acad. Sci..

[23] Meglasson MD (1993). Antihyperglycemic action of guanidinoalkanoic acids: 3-guanidinopropionic acid ameliorates hyperglycemia in diabetic KKAy and C57BL6Job/ob mice and increases glucose disappearance in rhesus monkeys. J. Pharmacol. Exp. Ther..

[24] Pandke KE, Mullen KL, Snook LA, Bonen A, Dyck DJ (2008). Decreasing intramuscular phosphagen content simultaneously increases plasma membrane FAT/CD36 and GLUT4 transporter abundance. Am. J. Physiol.-Regulatory Integr. Comp. Physiol..

[25] Jinnai D, Sawai A, Mori A (1966). γ-Guanidinobutyric acid as a convulsive substance. Nature.

[26] Mizuno A, Mukawa J, Kobayashi K, Mori A (1975). Convulsive activity of taurocyamine in cats and rabbits. IRCS Med. Sci.

[27] Bowery NG, Brown DA (1974). Depolarizing actions of γ-aminobutyric acid and related compounds on rat superior cervical ganglia in vitrO. Br. J. Pharmacol..

[28] Li YP, Lombardini JB (1990). Guanidinoethanesulfonic acid–inhibitor of GABA uptake in rat cortical synaptosomes. Brain Res..

[29] Mellor JR, Gunthorpe MJ, Randall AD (2000). The taurine uptake inhibitor guanidinoethyl sulphonate is an agonist at gamma-aminobutyric acid(A) receptors in cultured murine cerebellar granule cells. Neurosci. Lett..

[30] Herranz AS (1990). The epileptogenic action of the taurine analogue guanidinoethane sulfonate may be caused by a blockade of GABA receptors. J. Neurosci. Res..

[31] Sergeeva OA, Chepkova AN, Haas HL (2002). Guanidinoethyl sulphonate is a glycine receptor antagonist in striatum. Br. J. Pharmacol..

[32] Irreverre F, Evans RL, Hayden AR, Silber R (1957). Occurrence of gamma-guanidinobutyric acid. Nature.

[33] Kato T, Yamagata M, Tsukahara S (1986). Guanidine compounds in fruit trees and their seasonal variations in Citrus (*Citrus unshiu* Marc.). J. Jpn. Soc. Hortic. Sci..

[34] Popkov VA (2021). Gut microbiota as a source of uremic toxins. Int. J. Mol. Sci.

[35] Orellana MS (2022). New insights into the determinants of specificity in human type I Arginase: Generation of a mutant that is only active with Agmatine as substrate. Int. J. Mol. Sci..

[36] Lee SJ (2011). Crystal structures of *Pseudomonas aeruginosa* guanidinobutyrase and guanidinopropionase, members of the ureohydrolase superfamily. J. Struct. Biol..

[37] Kim DE, Chivian D, Baker D (2004). Protein structure prediction and analysis using the Robetta server. Nucleic Acids Res..

[38] Di Costanzo L (2005). Crystal structure of human arginase I at 1.29-Å resolution and exploration of inhibition in the immune response. Proc. Natl. Acad. Sci. U.S.A..

[39] Fukasawa Y (2015). MitoFates: Improved prediction of mitochondrial targeting sequences and their cleavage sites. Mol. Cell. Proteom..

[40] Wishart DS (2018). HMDB 4.0: The human metabolome database for 2018. Nucleic Acids Res..

[41] Uda K, Saishoji N, Ichinari S, Ellington WR, Suzuki T (2005). Origin and properties of cytoplasmic and mitochondrial isoforms of taurocyamine kinase. FEBS J..

[42] Yano D, Suzuki T (2021). Phosphagen kinases from five groups of eukaryotic protists (Choanomonada, Alveolate, Stramenopiles, Haptophyta, and Cryptophyta): Diverse enzyme activities and phylogenetic relationship with metazoan enzymes. Comp. Biochem. Physiol. B Biochem. Mol. Biol..

[43] Dawson DM, Eppenberger HM, Kaplan NO (1965). Creatine kinase: Evidence for a dimeric structure. Biochem. Biophys. Res. Commun..

[44] Schlegel J (1988). Mitochondrial creatine kinase from cardiac muscle and brain are two distinct isoenzymes but both form octameric molecules. J. Biol. Chem..

[45] Watanabe Y, Van Pilsum JF, Yokoi I, Mori A (1994). Synthesis of neuroactive guanidino compounds by rat kidney L-arginine: Glycine amidinotransferase. Life Sci..

[46] Hernández VM, Arteaga A, Dunn MF (2021). Diversity, properties and functions of bacterial arginases. FEMS Microbiol. Rev..

[47] Pisano JJ, Mitoma C, Udenfriend S (1957). Biosynthesis of γ-guanidinobutyric acid from γ-aminobutyric acid and Arginine. Nature.

[48] Huxtable RJ (1992). Physiological actions of taurine. Physiol. Rev..

[49] Holt A, Baker GB (1995). Metabolism of agmatine (clonidine-displacing substance) by diamine oxidase and the possible implications for studies of imidazoline receptors. Prog. Brain Res..

[50] Elmore BO, Bollinger JA, Dooley DM (2002). Human kidney diamine oxidase: Heterologous expression, purification and characterization. J. Biol. Inorg. Chem..

[51] Liu QR, Lopez-Corcuera B, Nelson H, Mandiyan S, Nelson N (1992). Cloning and expression of a cDNA encoding the transporter of taurine and beta-alanine in mouse brain. Proc. Natl. Acad. Sci. U. S. A..

[52] Huxtable RJ, Laird HE, Lippincott SE (1979). The transport of taurine in the heart and the rapid depletion of tissue taurine content by guanidinoethyl sulfonate. J. Pharmacol. Exp. Ther..

[53] Celik VK (2017). Serum levels of polyamine synthesis enzymes increase in diabetic patients with breast cancer. Endocr. Connect..

[54] Dallmann K (2004). Human agmatinase is diminished in the clear cell type of renal cell carcinoma. Int. J. Cancer.

[55] Chen X (2010). Novel association strategy with copy number variation for identifying new risk loci of human diseases. PLoS One.

[56] Zhu HE, Yin JY, Chen DX, He S, Chen H (2019). Agmatinase promotes the lung adenocarcinoma tumorigenesis by activating the NO-MAPKs-PI3K/Akt pathway. Cell Death Dis..

[57] Snezhkina AV (2016). The dysregulation of polyamine metabolism in colorectal cancer is associated with overexpression of c-Myc and C/EBPbeta rather than enterotoxigenic *Bacteroides fragilis* infection. Oxid. Med. Cell Longev..

[58] Fagerberg L (2014). Analysis of the human tissue-specific expression by genome-wide integration of transcriptomics and antibody-based proteomics. Mol. Cell. Proteom..

[59] Duff MO (2015). Genome-wide identification of zero nucleotide recursive splicing in Drosophila. Nature.

[60] Bernstein HG (2012). Agmatinase, an inactivator of the putative endogenous antidepressant agmatine, is strongly upregulated in hippocampal interneurons of subjects with mood disorders. Neuropharmacology.

[61] Al-Khelaifi F (2019). Metabolic GWAS of elite athletes reveals novel genetically-influenced metabolites associated with athletic performance. Sci. Rep..

[62] Wang J, Wang W, Kollman PA, Case DA (2006). Automatic atom type and bond type perception in molecular mechanical calculations. J. Mol. Graph. Model..

[63] Maier JA (2015). ff14SB: Improving the accuracy of protein side chain and backbone parameters from ff99SB. J. Chem. Theory Comput..

[64] Wang J, Wolf RM, Caldwell JW, Kollman PA, Case DA (2004). Development and testing of a general amber force field. J. Comput. Chem..

[65] Trott O, Olson AJ (2010). AutoDock Vina: Improving the speed and accuracy of docking with a new scoring function, efficient optimization and multithreading. J. Comput. Chem..

[66] Geyer JW, Dabich D (1971). Rapid method for determination of arginase activity in tissue homogenates. Anal. Biochem..

[67] Forlani G, Funck D (2020). A specific and sensitive enzymatic assay for the quantitation of L-proline. Front. Plant Sci..

[68] Bates LS, Waldren RP, Teare ID (1973). Rapid determination of free proline for water-stress studies. Plant Soil.

