# Supplementary material for: Guanidino acid hydrolysis by the human enzyme annotated as agmatinase
Source: Sci Rep. 2022 Dec 21;12:22088. doi: 10.1038/s41598-022-26655-4 (PMC9772407; doi:10.1038/s41598-022-26655-4)
Supplement: Supplementary file 1 — Supplementary Information. [file 41598_2022_26655_MOESM1_ESM.docx]

Supplementary Material for

**Guanidino acid hydrolysis by the human enzyme annotated as agmatinase**

Malte Sinn^1^, Marco Stanoppi^1^, Franziskus Hauth^1,2^, Jennifer R. Fleming^3^, Dietmar Funck^1^, Olga Mayans^2,3^, and Jörg S. Hartig ^1,2^

^1^ Department of Chemistry, University of Konstanz, Germany

^2^ Konstanz Research School Chemical Biology (KoRS-CB), University of Konstanz, Germany

^3^ Department of Biology, University of Konstanz, Germany

Content:

Supplementary Fig. S1

Supplementary Fig. S2

Supplementary Fig. S3

Supplementary Fig. S4

Synthesis and Characterization of guanidine compounds


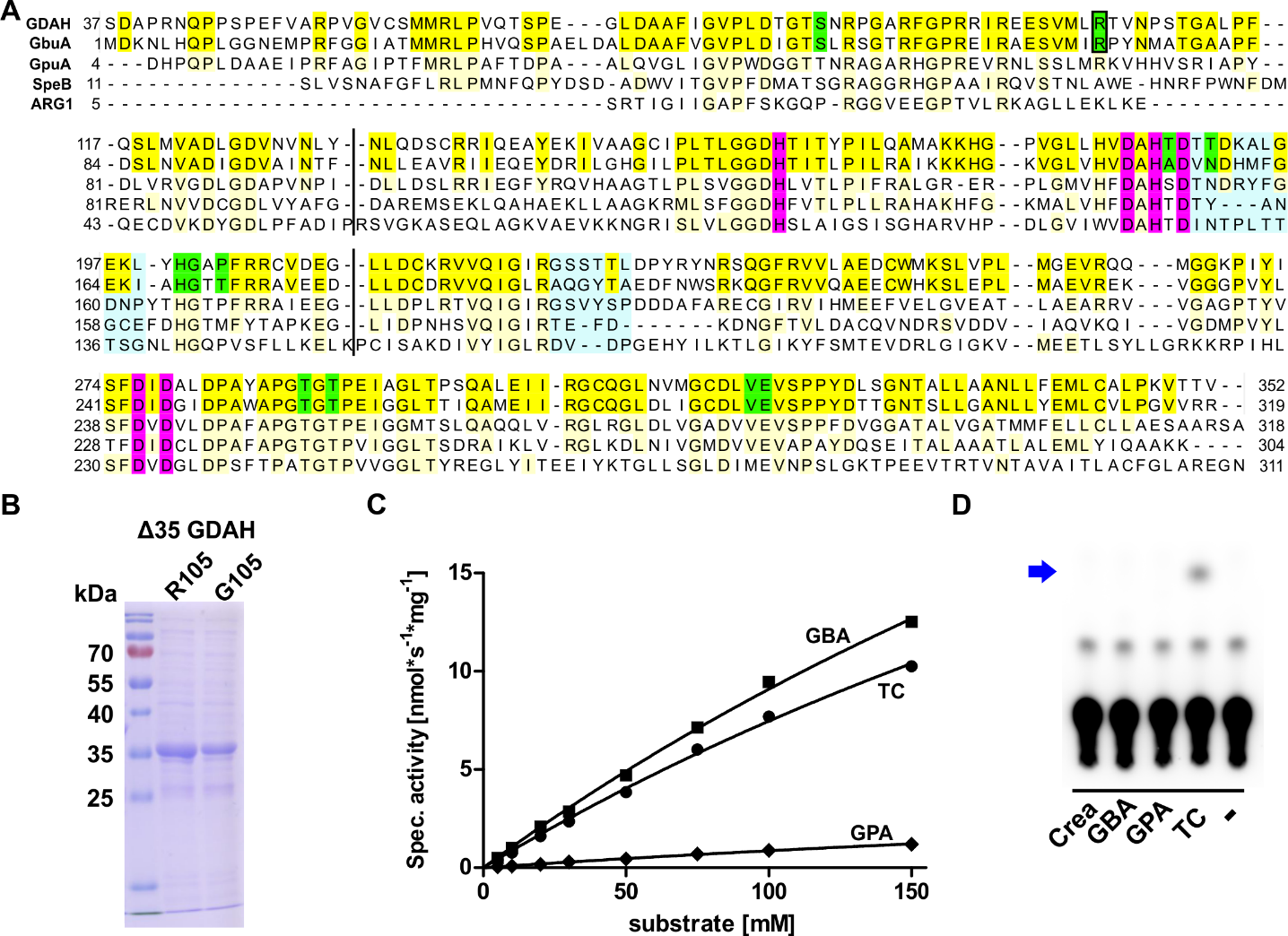


Supplementary Fig. S1: Multiple sequence alignment, purification and kinetics of GDAH and activity of taurocyamine kinase. A Multiple sequence alignment of human guanidino acid hydrolase GDAH with guanidinobutyrase GbuA and guanidinopropionase GpuA from *P. aeruginosa*, agmatinase SpeB from *E. coli* and human arginase ARG1. Conserved residues of GDAH and GbuA are marked with yellow. Residues of the other enzymes are marked (light yellow) if identical to the conserved residues of GDAH and GbuA. Metal coordination sites are highlighted in pink. Residues within 5Å of the modeled substrate (Fig. 2A) are highlighted in green. R105 of GDAH and R72 of GbuA that are approaching the binding pocket from a neighboring subunit are boxed. The two loops that mediate substrate specificity in SpeB and Arg1 are highlighted in blue. Black lines designate deletion of parts of the ARG1 sequence that do not align to the other sequences. B Coomassie-blue stained SDS -gel of purified recombinat GDAH. Variants R105 and G105 both run at approximately 35 kDA when the mitochondrial transit peptide (residues 1-35) is replaced by a 6xHis-tag and TEV cleavage site. C The specific activity of GDAH variant G105 was determined for different substrate concentrations of TC, GBA and GPA. Means of technical triplicates were plotted against the concentration with error bars representing s.d.. Data were fitted with Michaelis-Menten kinetics. D Radiograph of the phosphorylation reaction of the TC kinase from *A. brasiliensis*. Purified recombinant TC kinase was incubated with γ-^32^P-ATP and 10 mM of the respective substrate (creatine (crea), GBA, GPA, TC and no substrate (-)). Reaction products were separated by TLC as described in the methods. A phosphorylation product was only observed for TC (blue arrow). Additional bands arise also in the control reaction and are not enzyme or substrate related.


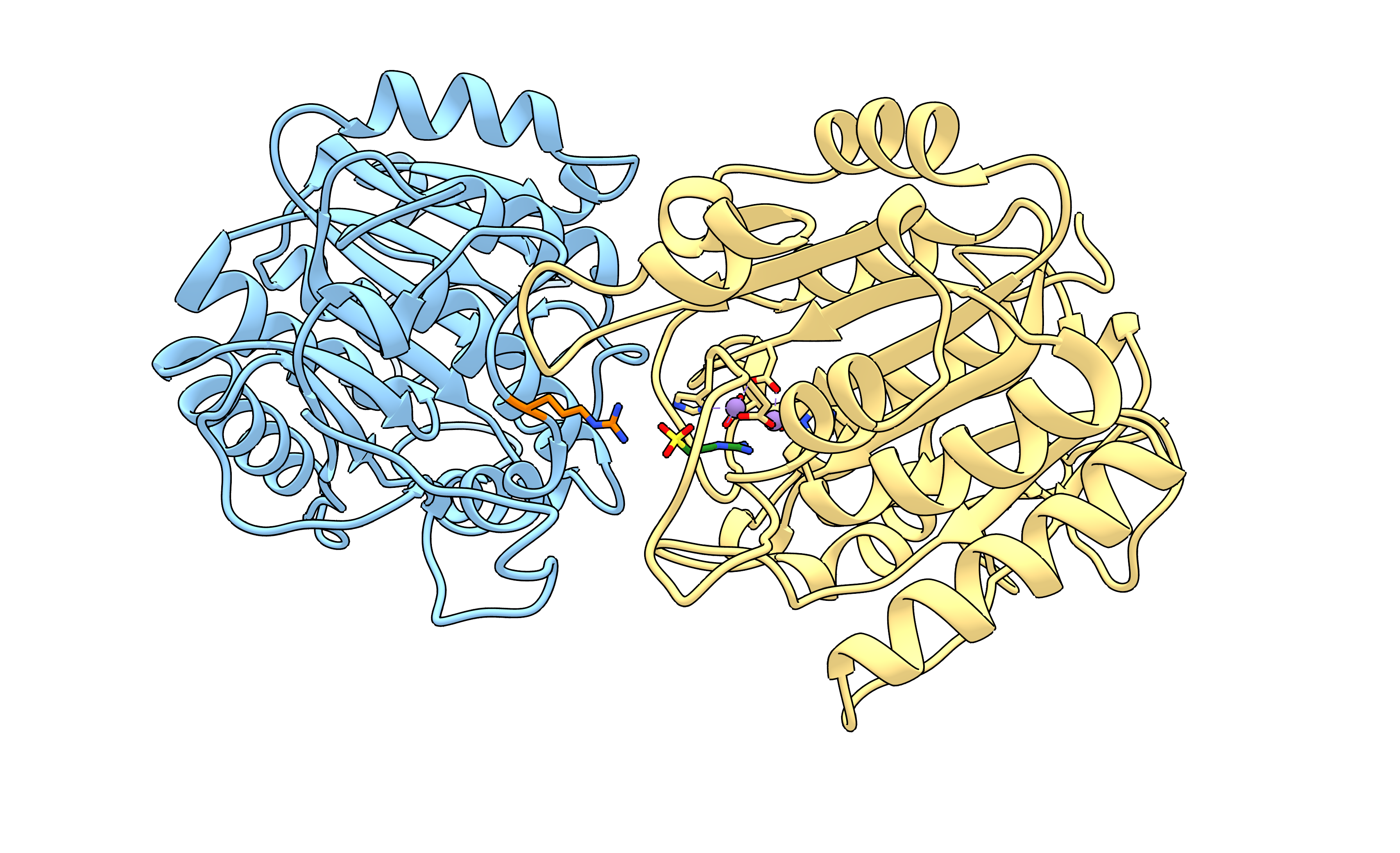


Supplementary Fig. S2: Global view of the GDAH model. Two subunits of GDAH were modeled on GbuA (3NIO) showing the catalytic site at the interface between the two subunits. R105 reaches from the neighboring subunit towards the substrate. Side chains of the residues coordinating the metal ions are depicted as sticks. R105 reaching from the neighboring subunit into the substrate binding pocket is highlighted in orange and the substrate taurocyamine in green


Supplementary Fig. S3: Representative calibration curve for urea determination. Standards were prepared for each experiment in the range of app. 2 µM-250 µM urea. Absorption of the respective substrate control was subtracted from the absorption of the samples before determination of the produced urea in the assay.


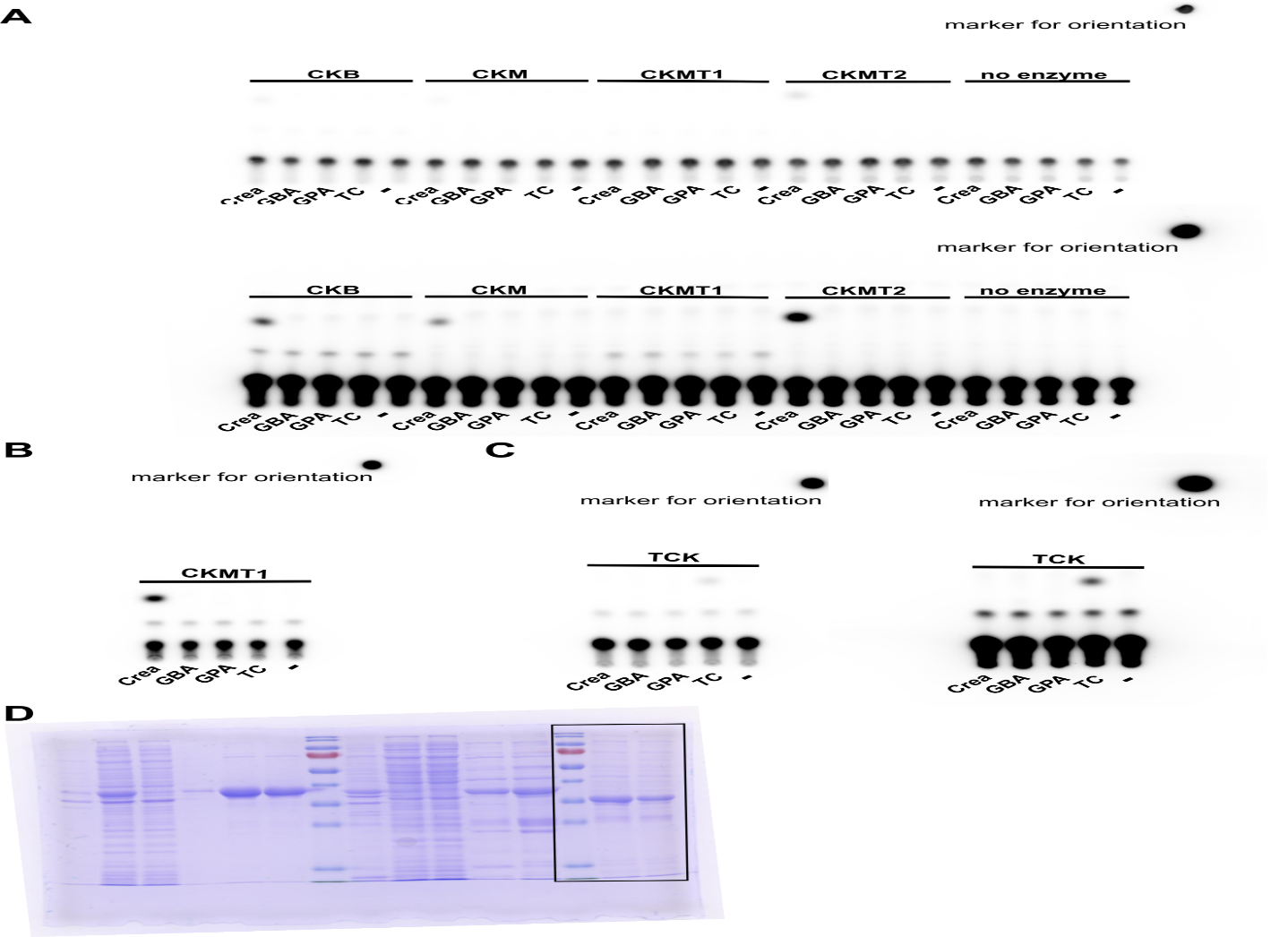


Supplementary Fig. S4: Original data of radiographs and pictures. A Low contrast (upper picture) and high contrast (lower picture) picture of the radiograph of the TLC plate shown in Figure 4A except for the reaction of CKMT1. B Radiograph of the TLC plate for the reaction with CKMT1 that was overlaid with the radiograph in A for Figure 4. C Low contrast (left picture) and high contrast (right picture) of the TLC plate for TCK reaction shown in Supplementary Fig.1D. D Original picture of the SDS gel for GDAH variant purification. Black box depicts the segment shown in Supplementary Fig.1B.

# Synthesis of guanidine compounds

## GPS sulfate

**Synthesis**

3-Amino-1-propanesulfonic acid (0.100 g, 0.720 mmol) was dissolved in distilled water (0.58 mL) and concentrated ammonia (25%, 0.116 mL, 1.55 mmol) was added. Solid *S*-methylisothiourea sulfate (0.110 g, 0.79 mmol) was then added portion-wise over the course of 3 min and the mixture stirred for 16 h. Note, the reaction should be performed in a well-ventilated fume hood due to the evolution of methanethiol. The reaction mixture was then freeze dried and the obtained solid was purified on acidic ion exchange resin. The by-products were initially eluted with distilled water. 1 M ammonia solution is then used as the eluent to recover the desired material obtained as a white solid after freeze drying the corresponding fractions (0.056 g, 0.245 mmol, 34% yield)

^1^H NMR (500 MHz, D_2_O): δ 3.37 (2H, td, *J* = 1.1 Hz, *J* = 6.9 Hz), 3.37 (2H, td, *J* = 1.2 Hz, *J* = 7.7 Hz), 3.37 (2H, p, *J* = 7.14 Hz).

^13^C{^1^H} NMR (126 MHz, D_2_O): δ 156.96, 47.98, 39.73,23.79.

**NMR Spectra**


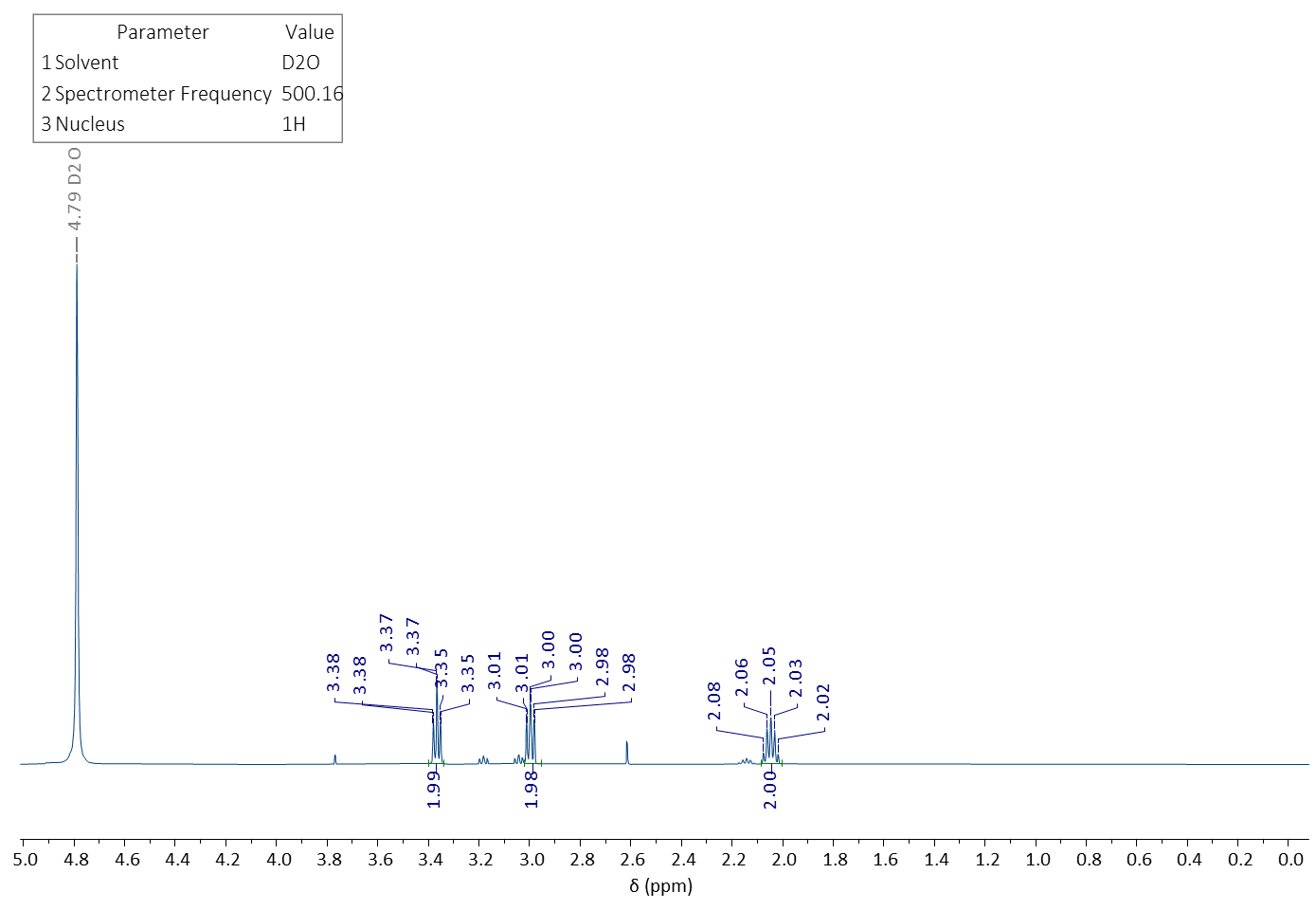


Supplementary Fig. S3: ^1^H NMR of GPS sulfate


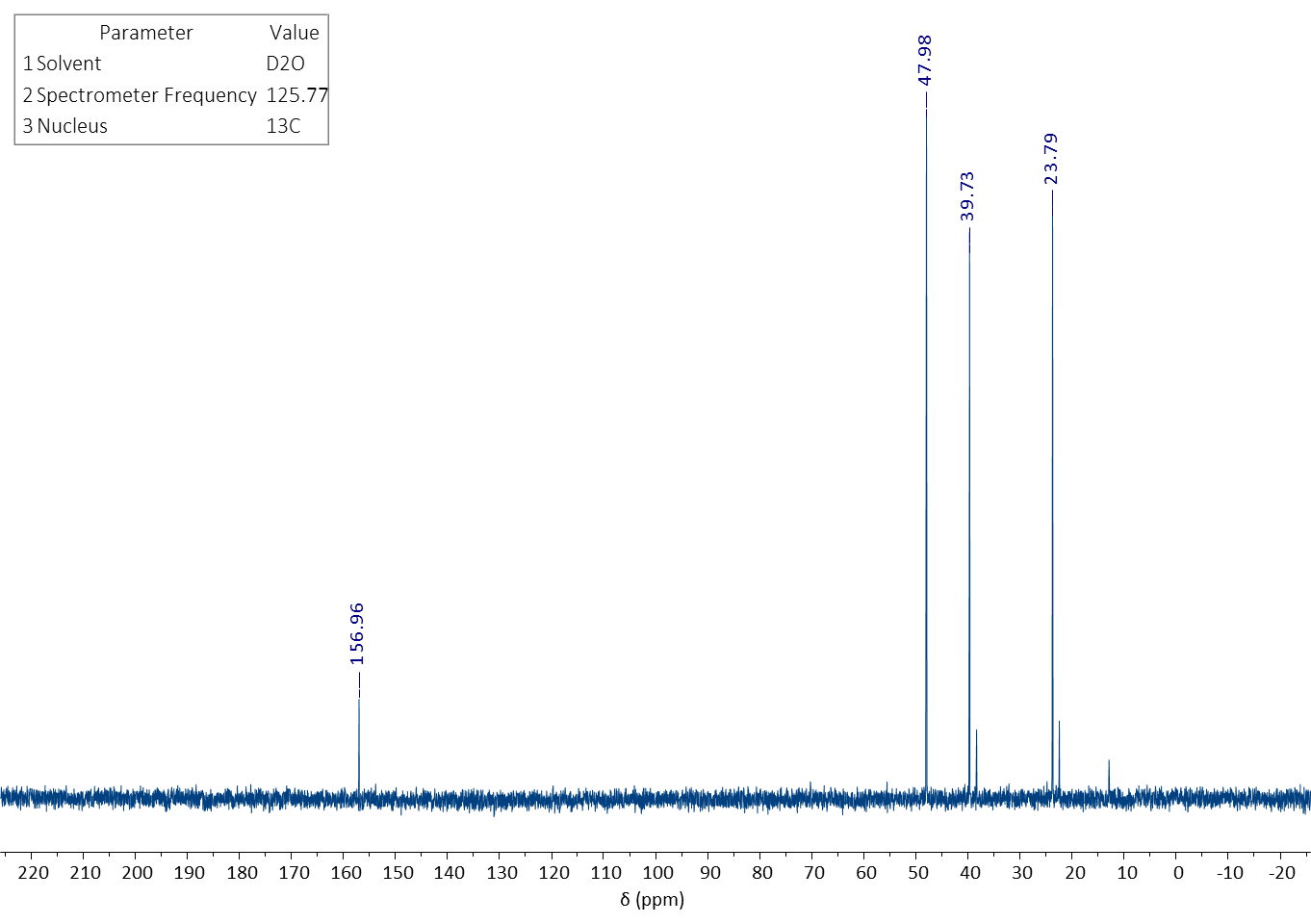


Supplementary Fig. S4: ^13^C NMR of GPS sulfate

## Hypo-TC

**Synthesis**

The target compound was synthesized according to the procedure reported by Desvages and van Thoai^1^. The NMR signal recorded for the obtained compound corresponded to the one reported in the literature.

**NMR Spectra**


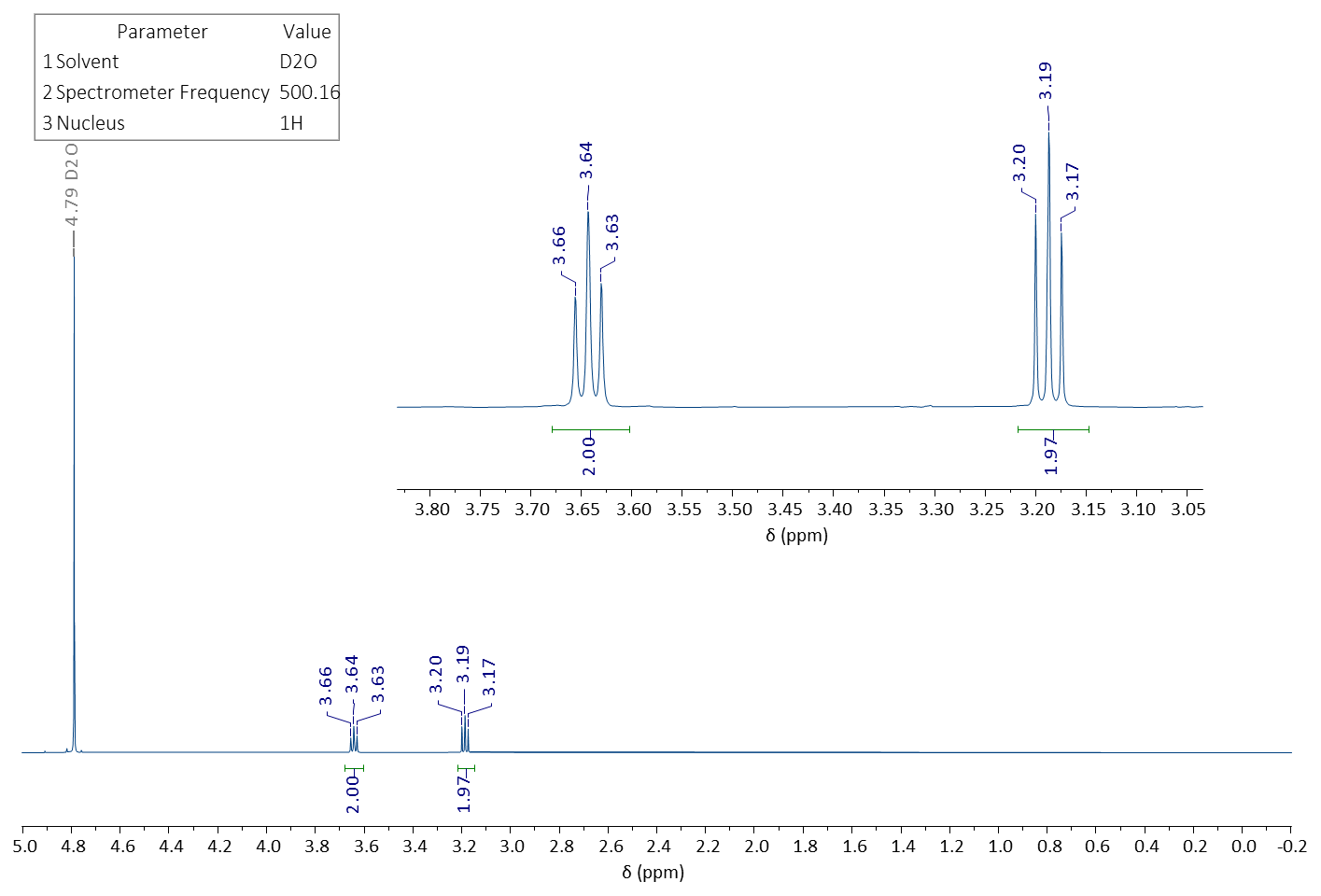


Supplementary Fig. S5: ^1^H NMR of Hypo-TC


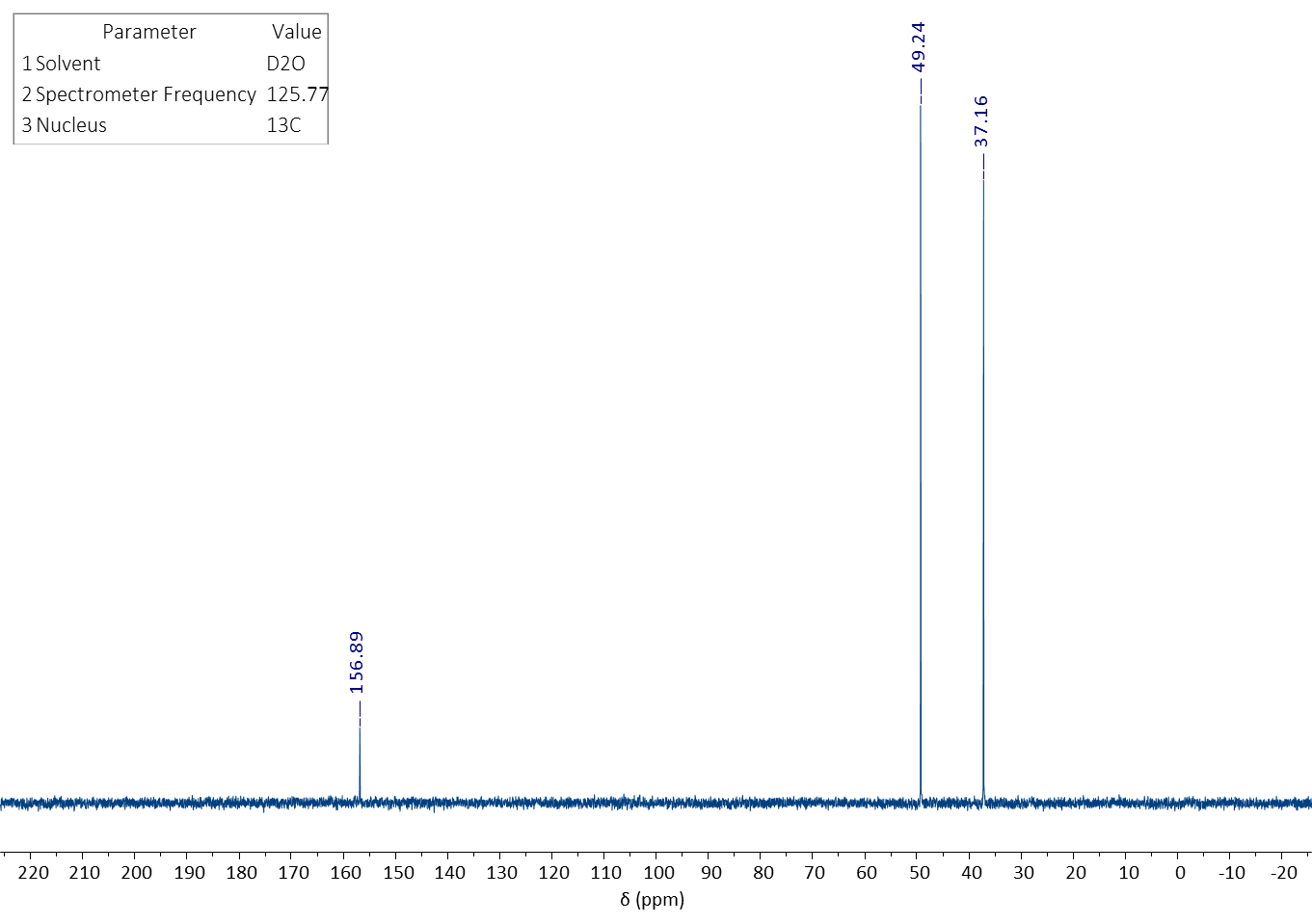


Supplementary Fig. S6: ^13^C NMR of Hypo-TC

## Gd-EtOH sulfate

**Synthesis**

Gd-EtOH was synthesized using a modified version of the procedure reported by Burda and Rademann^2^. Ethanolamine (0.118 mL, 1.96 mmol) and *S*-isomethylthiourea sulfate ( 0.273 g, 1.96 mmol) were added in a round bottom flask and ethanol (3.6 mL) was added. The mixture was heated for 6 h at 85 °C and then the solvent was evaporated. The obtained oil crystalized upon storage at 5 °C, the solid was then washed with cold ethanol (3x 0.5 mL) and dried under vacuum. The desired product was obtained as a white solid (0.120 g, 0.789 mmol, 40 % yield).

^1^H NMR (500 MHz, D_2_O): δ 3.74 (2H, t, *J* = 4.8 Hz), 3.37 (2H, t, *J* = 4.9 Hz).

^13^C{^1^H} NMR (126 MHz, D_2_O): δ 157.45, 59.90, 43.45.

**NMR Spectra**

**
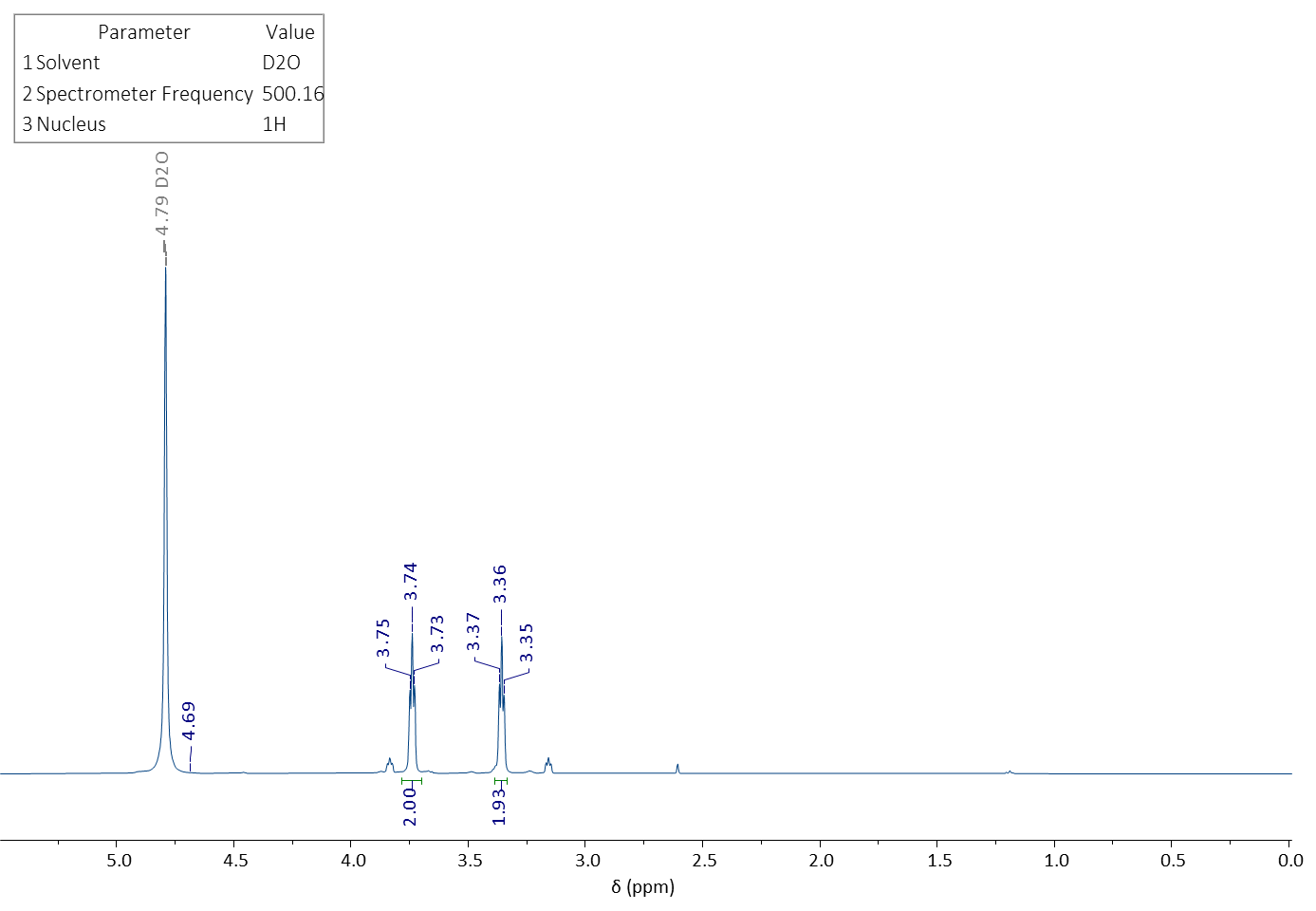
**

Supplementary Fig. S7: ^1^H NMR of Gd-EtOH sulfate


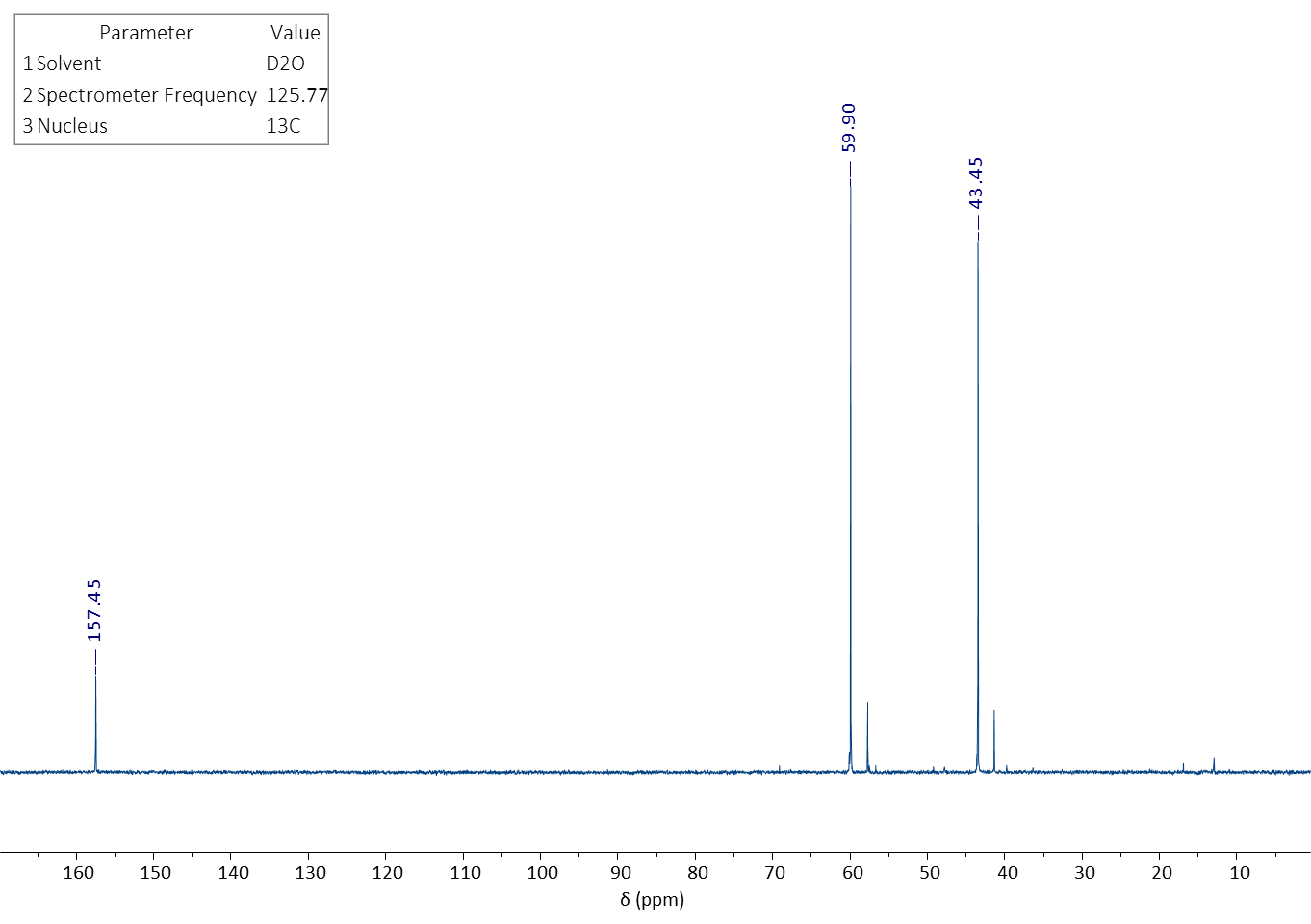


Supplementary Fig. S8: ^13^C NMR of Gd-EtOH sulfate

## MEG sulfate

**Synthesis**

MEG sulfate was synthesised using the same procedure described for GPS sulfate. For its synthesis Cysteamine hydrochloride (2.0 g, 17.6 mmol), *S*-methylisothiourea sulfate (2.7 g, 19.4 mmol) concentrated ammonia (25%, 2.8 mL, 37.7 mmol) and water (14 mL) were used. The product was obtained as a white solid after purification (1.08 g, 9.06 mmol, 51% yield).

^1^H NMR (500 MHz, D_2_O): δ 3.25 (2H, t, *J* = 7.1 Hz), 2.64 (2H, t, *J* = 7.1 Hz).

^13^C{^1^H} NMR (126 MHz, D_2_O): δ 156.81, 46.60, 23.61.

**NMR Spectra**


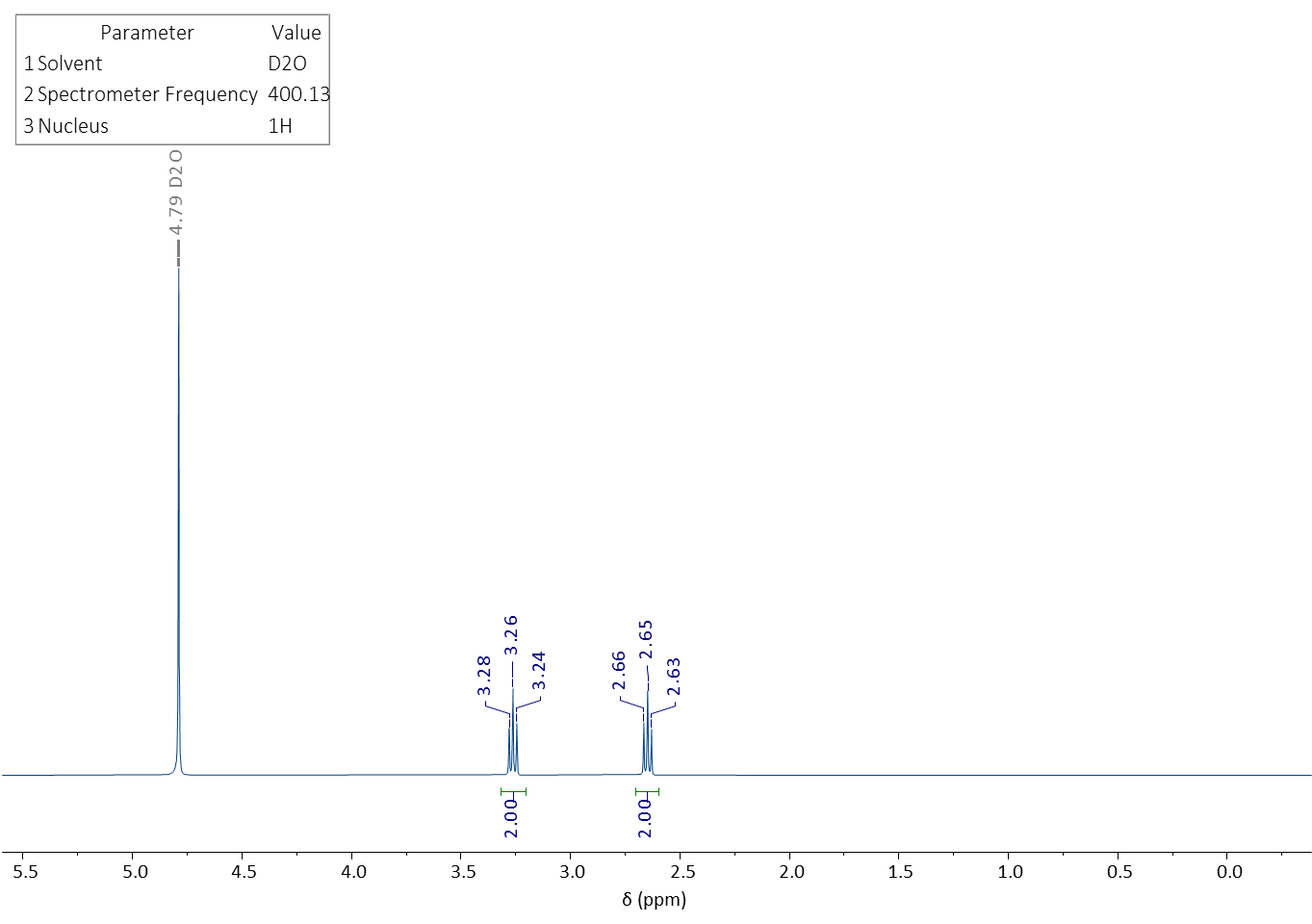


Supplementary Fig. S9: ^1^H NMR of MEG sulfate


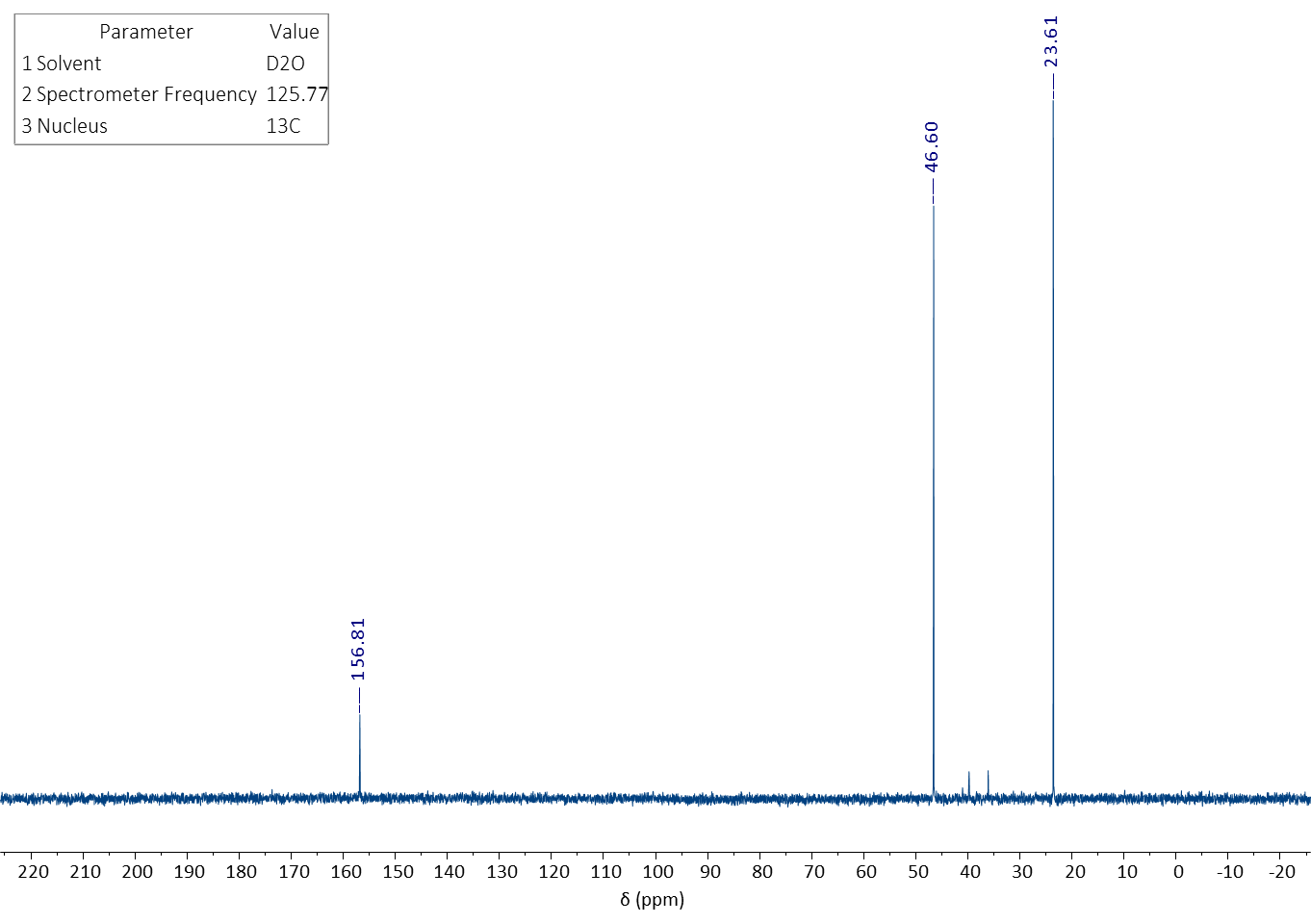


Supplementary Fig. S10: ^13^C NMR of MEG sulfate

## GSA

**Synthesis**

Aspartic acid (0.2 g, 1.5 mmol) was dissolved in a concentrated aqueous solution of ammonia (25%, 1 mL). *S*-methylisothiourea was then added to the solution portion-wise over 10 min and the reaction stirred for 16 h. Over the time a white precipitated formed and it was then separated from the suspension *via* centrifugation. The solid was then dissolved in distilled water (0.4 mL) and precipitated by adding ethanol (1 mL). The precipitate was then separated *via* centrifugation and dried under vacuum delivering the desired product as a white solid (0.140 g, 0.729 mmol, 49% yield).

^1^H NMR (500 MHz, D_2_O): δ 4.24 (2H, dd, *J* = 9.4 Hz, *J* = 3.5 Hz), 2.82c (2H, dd, *J* = 16.2 Hz, *J* = 3.5 Hz), 2.55 (2H, dd, *J* = 16.2 Hz, *J* = 9.4 Hz).

^13^C{^1^H} NMR (126 MHz, D_2_O): δ 178.78, 177.09, 156.87, 54.98, 40.53.

**NMR Spectra**


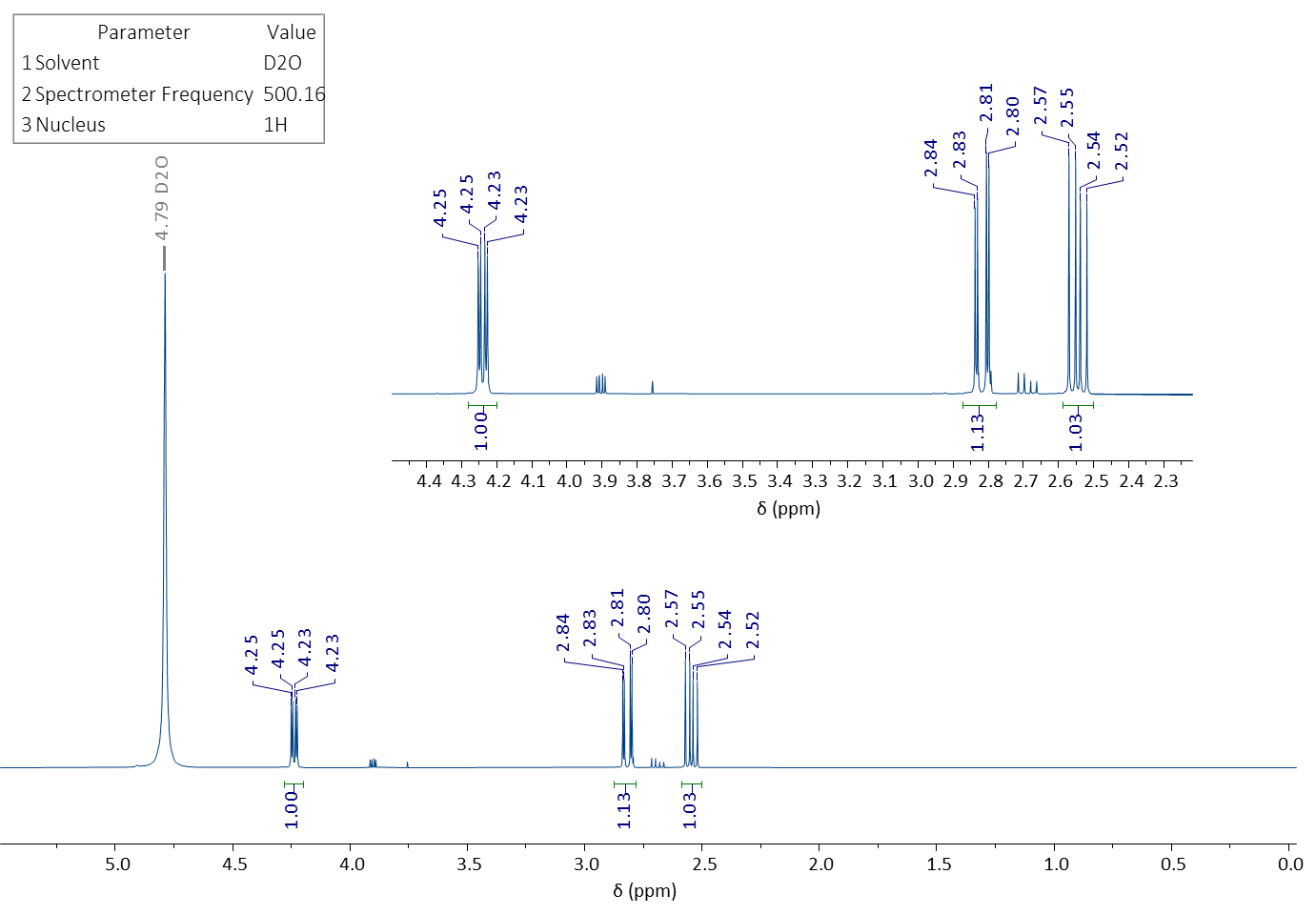


Supplementary Fig. S11: ^1^H NMR of GSA


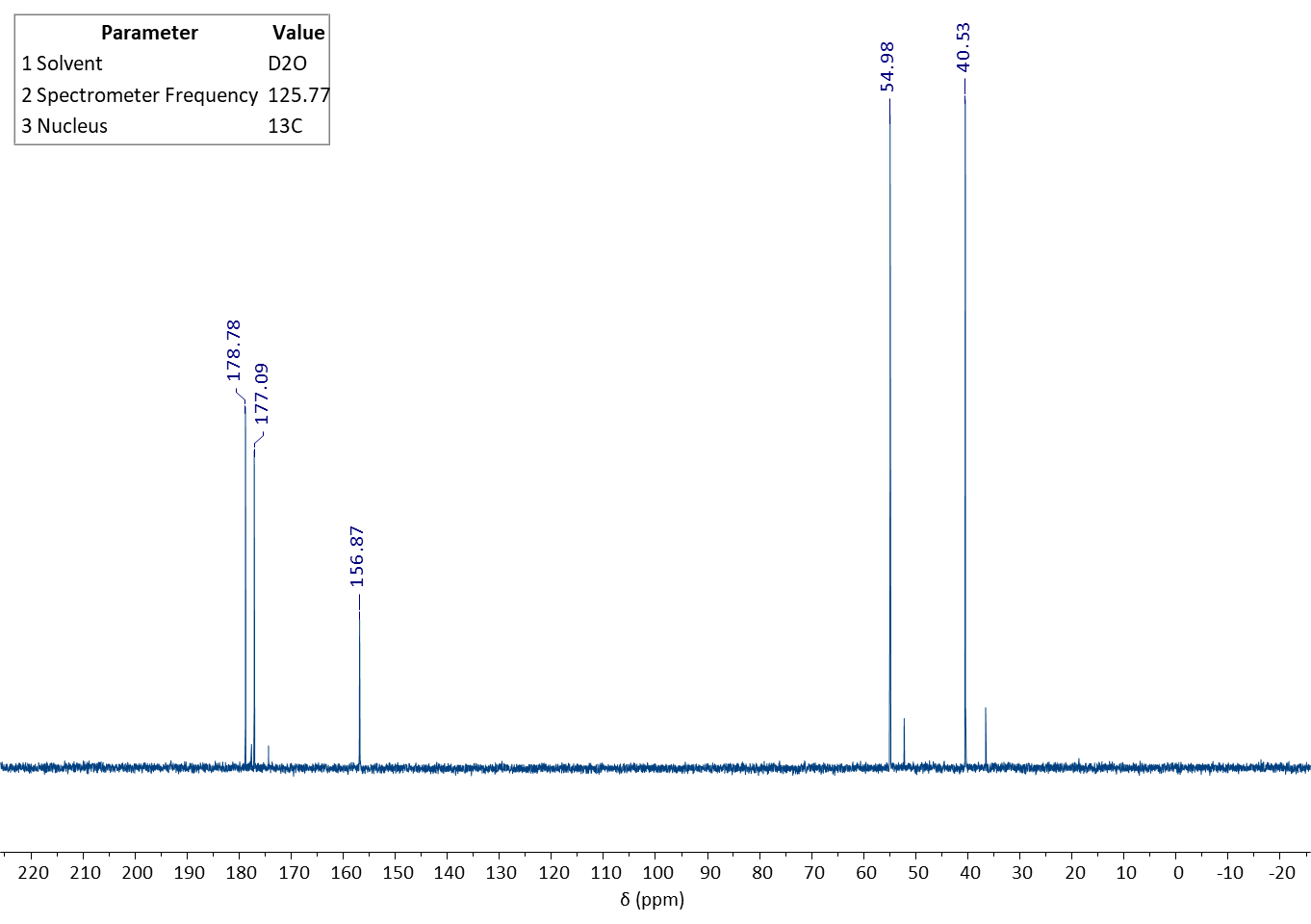


Supplementary Fig. S12: ^13^C NMR of GSA

**Additional References:**

1 Desvages, G. & van Thoai, N. Synthèse de l'hypotaurocyamine ou acide guanidino-2- éthane sulfinique. *C. R. Acad. Sc. Paris* **267**, 1868-1870 (1968).

2 Burda, E. & Rademann, J. Catalytic activation of pre-substrates via dynamic fragment assembly on protein templates. *Nature Communications* **5**, 5170 (2014). <https://doi.org:10.1038/ncomms6170>
